# The rises and falls of opsin genes in 59 ray-finned fish genomes and their implications for environmental adaptation

**DOI:** 10.1038/s41598-017-15868-7

**Published:** 2017-11-14

**Authors:** Jinn-Jy Lin, Feng-Yu Wang, Wen-Hsiung Li, Tzi-Yuan Wang

**Affiliations:** 1 Institute of Molecular and Cellular Biology, National Tsing Hua University, Hsinchu, 30013 Taiwan; 2 Bioinformatics Program, Taiwan International Graduate Program, Institute of Information Science, Academia Sinica, Nankang, Taipei, 11529 Taiwan; 3 Biodiversity Research Center, Academia Sinica, Nankang, Taipei, 11529 Taiwan; 4 Taiwan Ocean Research Institute, National Applied Research Laboratories, Kaohsiung, 852 Taiwan; 5 Department of Ecology and Evolution, University of Chicago, Chicago, 60637 USA

## Abstract

We studied the evolution of opsin genes in 59 ray-finned fish genomes. We identified the opsin genes and adjacent genes (syntenies) in each genome. Then we inferred the changes in gene copy number (*N*), syntenies, and tuning sites along each phylogenetic branch during evolution. The Exorh (rod opsin) gene has been retained in 56 genomes. Rh1, the intronless rod opsin gene, first emerged in ancestral Actinopterygii, and *N* increased to 2 by the teleost-specific whole genome duplication, but then decreased to 1 in the ancestor of Neoteleostei fishes. For cone opsin genes, the rhodopsin-like (Rh2) and long-wave-sensitive (LWS) genes showed great variation in *N* among species, ranging from 0 to 5 and from 0 to 4, respectively. The two short-wave-sensitive genes, SWS1 and SWS2, were lost in 23 and 6 species, respectively. The syntenies involving LWS, SWS2 and Rh2 underwent complex changes, while the evolution of the other opsin gene syntenies was much simpler. Evolutionary adaptation in tuning sites under different living environments was discussed. Our study provides a detailed view of opsin gene gains and losses, synteny changes and tuning site changes during ray-finned fish evolution.

## Introduction

Visual perception is important for an animal because it conveys the color, shape, size and movement of its surrounding objects. Based on the perceived information, an animal responds to changes in the surrounding environment.

The visual capability of an animal depends on the opsin genes it possesses. Opsins are G protein-coupled receptors, which are anchored to membrane via their transmembrane domains^1^. Phototransduction occurs via the interaction between opsin proteins and vitamin A-derived chromophore^2^. In vertebrates, there are five opsin gene families. In rod cells, rhodopsin genes (Rh1) are responsible for scotopic vision. There are two types of Rh1 genes: one possesses five exons and is denoted as Exorh, while the other has no intron and is known as intronless Rh1^3^. In cone cells, two short-wavelength sensitive (SWS1 and SWS2), one middle-wavelength sensitive (Rh2), and one long-wavelength sensitive (LWS) opsin genes are responsible for photopic vision. The interaction between an opsin and chromophore affects the maximum spectrum absorbance (λmax) of the opsin, which is the most important characteristic that defines its functionality. A change at a tuning site, a key residue on the interacting surface of an opsin and chromophore, can result in a shift in λmax^4–11^.

The diversity of habitats in the aqueous environment is a driving force of the evolution of opsin genes in fishes. For example, deep-sea fishes may receive only blue lights^12^ because long-wavelength lights carry lower energy and cannot travel to deep waters. Also, the turbidity of a water body affects penetration of UV-light^13^. Finally, the utilization of opsin genes may vary with life style, developmental stage^14^ or sex^15^.

Tandem duplication and whole genome duplication (WGD) have played important roles in the evolution of opsin genes in fishes^16,17^. It was hypothesized that the five opsin families were derived from duplication events in the genome of the common ancestor of vertebrates^16,17^. Fish genomes in different lineages have experienced two to four rounds of WGD^18–20^. The 2R (the second round of WGD) occurred prior to the divergence between the jawed and jawless vertebrates^17^. The 3R led to teleost-specific tetraploidization^19^. The 4R occurred once in the ancestor of common carps^20^ and once in the ancestor of Salmoniformes^18^. Most previous studies included only the 2R and 3R events because the number of available fish genomes was then limited^16,19^. Moreover, the evolutionary dynamics of opsin gene syntenies was not investigated in detail.

Phylogenetic analysis of fish opsin gene sequences has been conducted in sequenced fish genomes^21,22^ and the studies have been reviewed^16^. However, previously only the cichlid fishes had abundant synteny data for describing the evolutionary dynamics of opsin genes^23–25^. Among the opsin gene families, only SWS2 genes in Percomorpha fishes have been studied comprehensively^26^.

There are now about 76 fish genomes available^27^, so that a comprehensive study of the evolution of opsin genes in fish can be conducted. In this study, we mainly focused on ray-finned fishes. We selected 59 better assembled genomes and predicted their opsin genes, using our newly constructed bioinformatics pipeline. Second, we annotated the genomic locations and neighboring genes of opsin genes (syntenies). Third, we identified synteny changes and gains and losses of opsin genes in each branch of the phylogeny of ray-finned fishes. Fourth, we studied the evolutionary changes at major tuning sites. Finally, we discussed the implications of copy number changes and tuning site changes for the visual adaptation of ray-finned fishes dwelling in different habitats.

## Results

### Fish Phylogeny and Opsin Gene Prediction

**Figure 1:**
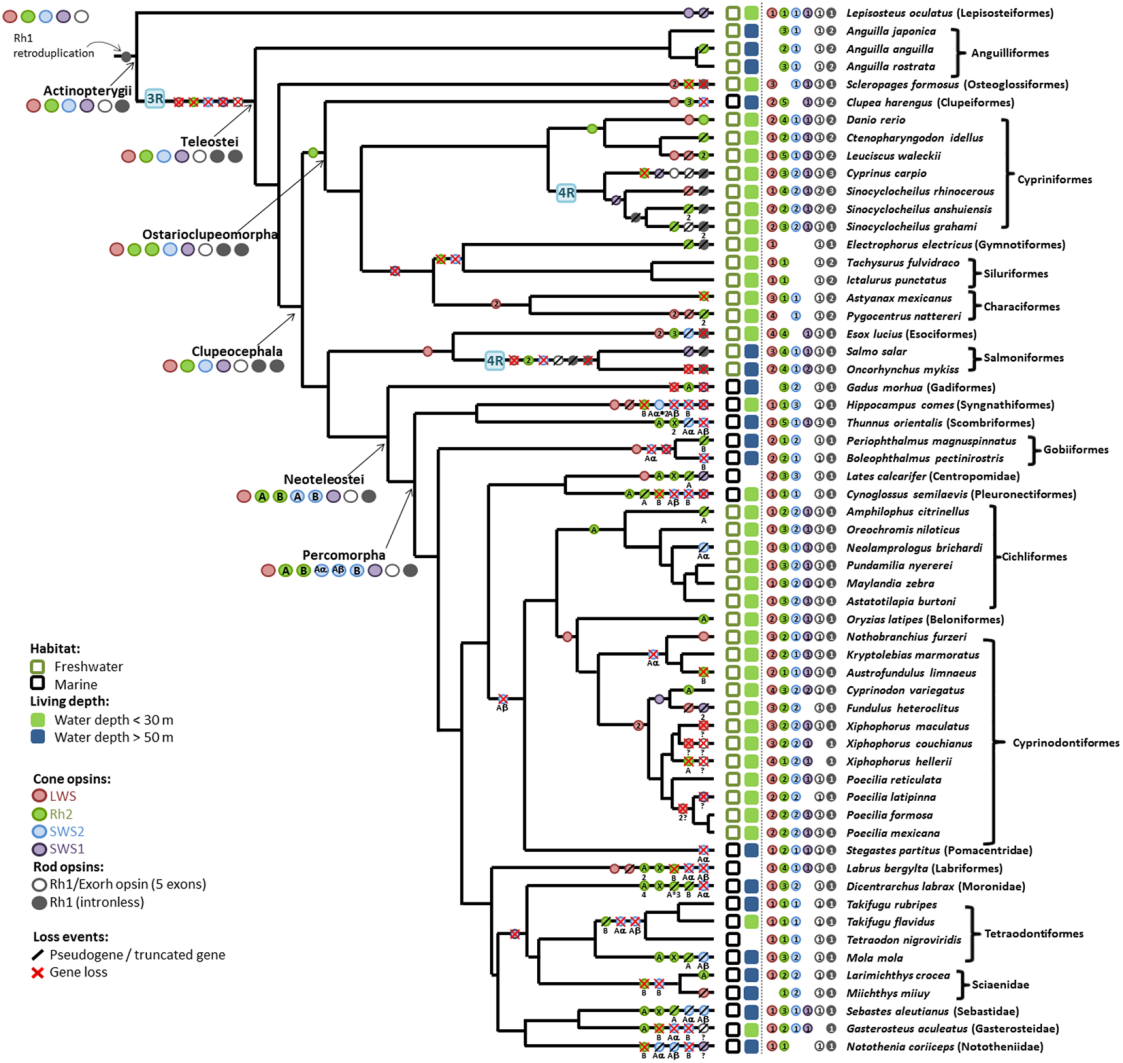
Fish cladogram and inferred opsin gene copy number changes in tree branches during fish evolution. The construction of the cladogram is described in the text. Color circles denote opsin gene symbols (LWS: red; SWS1: purple; SWS2: blue; Rh2: green; Rh1/Exorh (5 exons): gray hollow circle; and intronless Rh1: gray solid circle). Color squares denote the habitat symbols (freshwater: green open square; marine: black open square; living water depth <30 m: green solid square; living water depth >50 m: blue solid square) (Supplementary Table S1). The opsin genes found in an extant fish genome are shown before the species name in the order of LWS, Rh2, SWS2, SWS1, Rh1/Exorh, and Rh1 (intronless); if an opsin is missing in a species, the position in that species is empty (no circle). The inferred opsin genes in each major ancestral node are presented. An inferred opsin gene duplication event on a branch is indicated by its gene symbol on the branch; the number inside a circle indicates the number of gene gains. A pseudogenization event is indicated by a black slash, while a gene loss event is indicated by a red “X”. The pseudogenes are excluded when counting the total number of opsin genes in an extant fish genome. For Rh2 gene, a Rh2 gene symbol with black X indicates that the duplicated Rh2 gene experienced gene conversion even similar to what Nakamura *et al*.^21^ observed on some of the Rh2 genes of *Thunnus orientalis*. For a fish order/family that only has one species included in this study, the order/family name (in parentheses) and the name of the species are shown. In addition, a gene loss event that may have resulted from incomplete assembly of the genomic region is indicated by “?”. Note that a 4R event occurred in the ancestral genome of Salmoniformes and another occurred in the genome of the *Cyprinus carpio* (Cypriniformes).

We selected 59 better assembled genomes from 76 available ray-finned fish genomes (Fig. 1 and Supplementary Table S1). The data of their living environments were collected from FishBase^28^ (Supplementary Table S1). From a comprehensive molecular phylogeny of fishes^29^, TimeTree^30^ and the studies of Cyprinodontiformes^31^ and Cichliformes^23^, we obtained a cladogram of the 59 ray-finned fishes (Fig. 1). This cladogram was used to infer gene copy number changes and its relationship to environmental adaptation, and synteny changes.

Using the 59 genome assemblies and our bioinformatics pipeline (Materials and Methods, Supplementary Fig. S1), we identified 547 opsin genes in the 59 genomes (Supplementary Table S2). These 547 genes include 483 complete genes, 32 pseudogenes and 32 truncated genes (missing DNA segments $\geqq$100 bps); the pseudogenes and truncated genes were not included when counting the number of opsin genes in a genome. Figure 1 shows the types and numbers of opsin genes observed or predicted in each fish genome.

### Opsin Gene Copy Number Changes during Fish Evolution

We used the parsimony principle to infer the copy number of each type of opsin gene at each ancestral node and also the minimum gene copy number changes in each branch of the cladogram (Fig. 1). The minimum changes were then checked when we considered the synteny changes in each branch of the tree and modified an inferred number if it was not consistent with the inferred synteny changes. The phylogenetic trees of the five opsin gene families are shown in Supplementary Figs S2–S6. The evolution of copy number of each opsin gene family is shown in Supplementary Figs S7–S11. Our inferences are summarized below.

First, we inferred that all five types of opsin genes (LWS, Rh2, SWS1, SWS2, and Rh1 (Exorh)) already existed in the common ancestor of the 59 ray-finned fishes (Fig. 1) and a retro-duplication of the Rh1 gene occurred in the ancestor of ray-finned fishes because an intronless Rh1 gene exists in all ray-finned fishes^16^ (Fig. 1 and Supplementary Figs S7–S11). Second, the 3R event in the ancestral teleost should have duplicated all five types of opsin genes, but only one copy of each of the LWS, SWS1, SWS2, Rh2, and Exorh genes, and two intronless Rh1 genes were retained in the ancestral teleost genome (Fig. 1 and Supplementary Figs S7–S11). Third, one Rh2 gene duplication happened in Ostarioclupeomorpha (Fig. 1 and Supplementary Fig. S10). Fourth, the common ancestor of *Electrophorus electricus* (electric eel), *Astyanax mexicanus* (Mexican tetra), *Pygocentrus nattereri* (red-bellied piranha), *Tachysurus fulvidraco* (yellow catfish) and *Ictalurus punctatus* (channel catfish) may have lost its SWS1 gene (Fig. 1 and Supplementary Fig. S9). Subsequent gene gains and losses are shown in Fig. 1.

The 4R event in the ancestor of *Cyprinus carpio* and *Sinocyclocheilus* spp. fishes should have doubled the copy numbers of the then-existing opsin genes. The *C*. *carpio* lineage subsequently gained one Exorh genes but it also lost one Rh2 gene and had one SWS1 gene, two Exorh genes and one intronless Rh1 gene was truncated (Fig. 1 and Supplementary Fig. S12). In the *Sinocyclocheilus* lineage one of the SWS1 genes became a pseudogene (Supplementary Fig. S12). The common ancestor of *Oncorhynchus mykiss* (rainbow trout) and *Salmo salar* (Atlantic salmon) also underwent a 4R event, but *O*. *mykiss* and *S*. *salar* have subsequently lost many opsin genes (Supplementary Fig. S13).

The ancestral Neoteleostei genome lost one intronless Rh1 gene, but gained one SWS2 gene and one Rh2 gene (Fig. 1 and Supplementary Figs S8, S10 and S11). Then, the two Rh2 genes diverged into Rh2A and Rh2B with different λmax values (Rh2A: 500 to 530 nm, Rh2B: 450 to 500 nm)^4^. Also, the two SWS2 genes diverged into SWS2A and SWS2B (SWS2A: 440 to 460 nm, SWS2B: 400 to 420 nm)^32^. In the ancestor of Percomorpha, SWS2A was duplicated and diverged into Aα and Aβ, so that it possessed three SWS2 genes (SWS2Aα, SWS2Aβ and SWS2B) (Fig. 1 and Supplementary Fig. S8). In addition, it possessed one LWS gene, one SWS1 gene, two Rh2 genes, one Exorh gene, and one intronless Rh1 gene. Therefore, the extant Percomorpha species contain many types of opsin genes (Fig. 1).

### Evolution of Opsin Gene Syntenies

**Figure 2:**
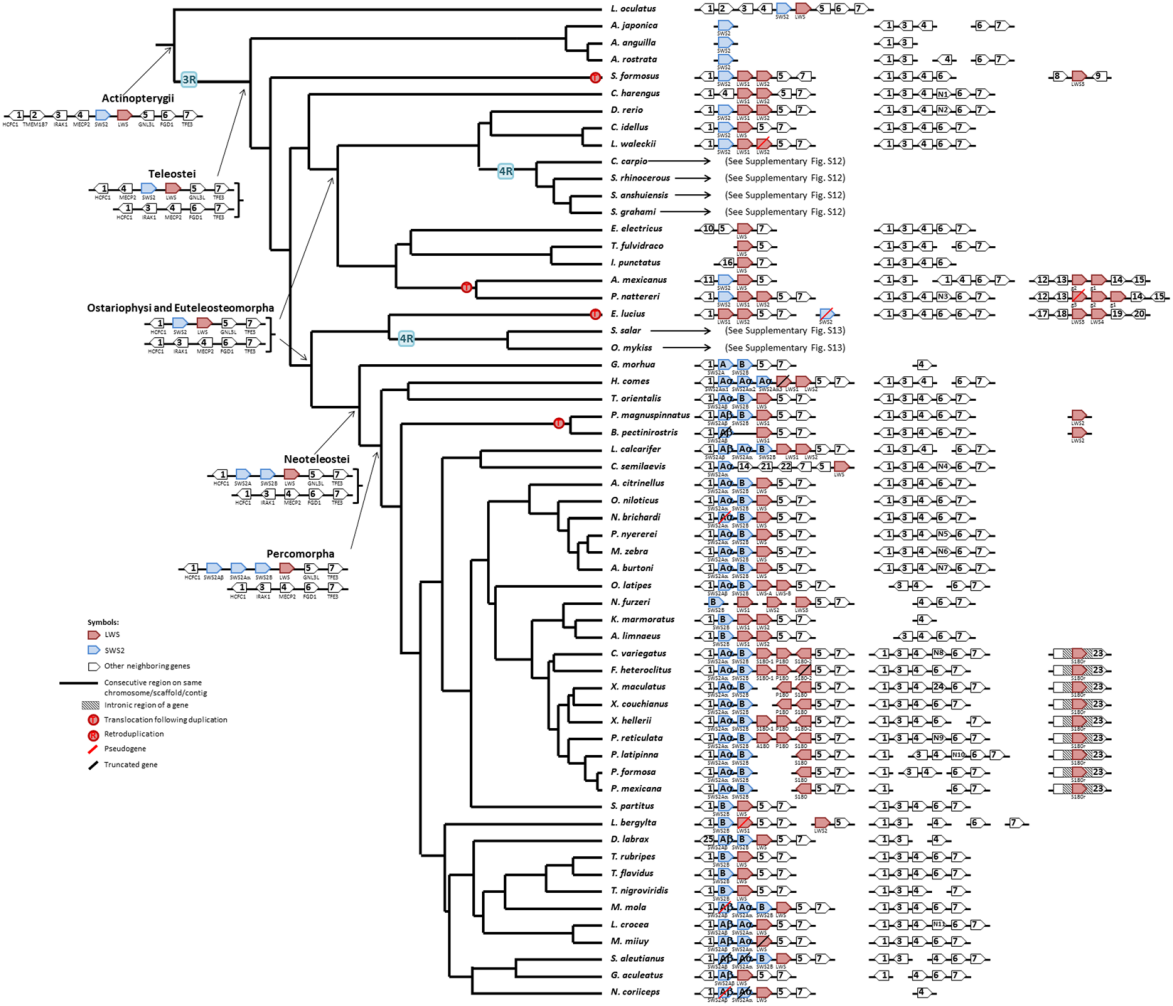
The most parsimonious tree of the SWS2-LWS syntenies. The syntenies in the genomes that experienced a 4R are summarized in Supplementary Fig. S12 (*Cyprinus carpio* and *Sinocyclocheilus spp*.) and S13 (Salmoniformes). The genome annotations we used are summarized in Supplementary Table S1. The genomic location of each synteny is summarized in Supplementary Table S4. Due to space constraint, only up two genes upstream and up two genes downstream of the opsin genes are shown for the cases in which a translocation or rearrangement of neighboring genes occurred. Each neighboring gene is denoted by a gene number or a gene ID if its function is unknown. The complete list of gene names and gene IDs is given in Supplementary Text.

**Figure 3:**
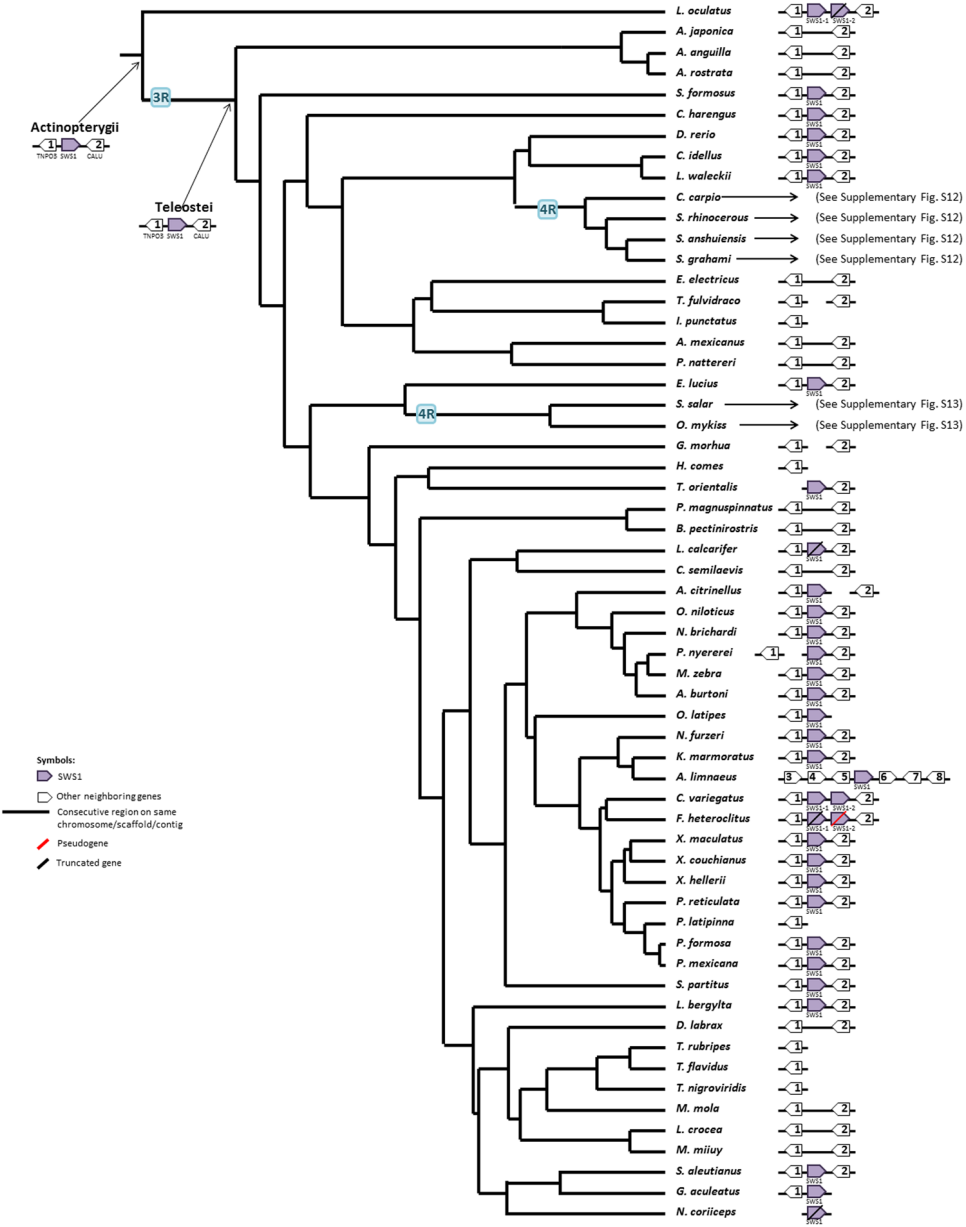
The most parsimonious tree of the SWS1 syntenies. See the legend of Fig. 2.

**Figure 4:**
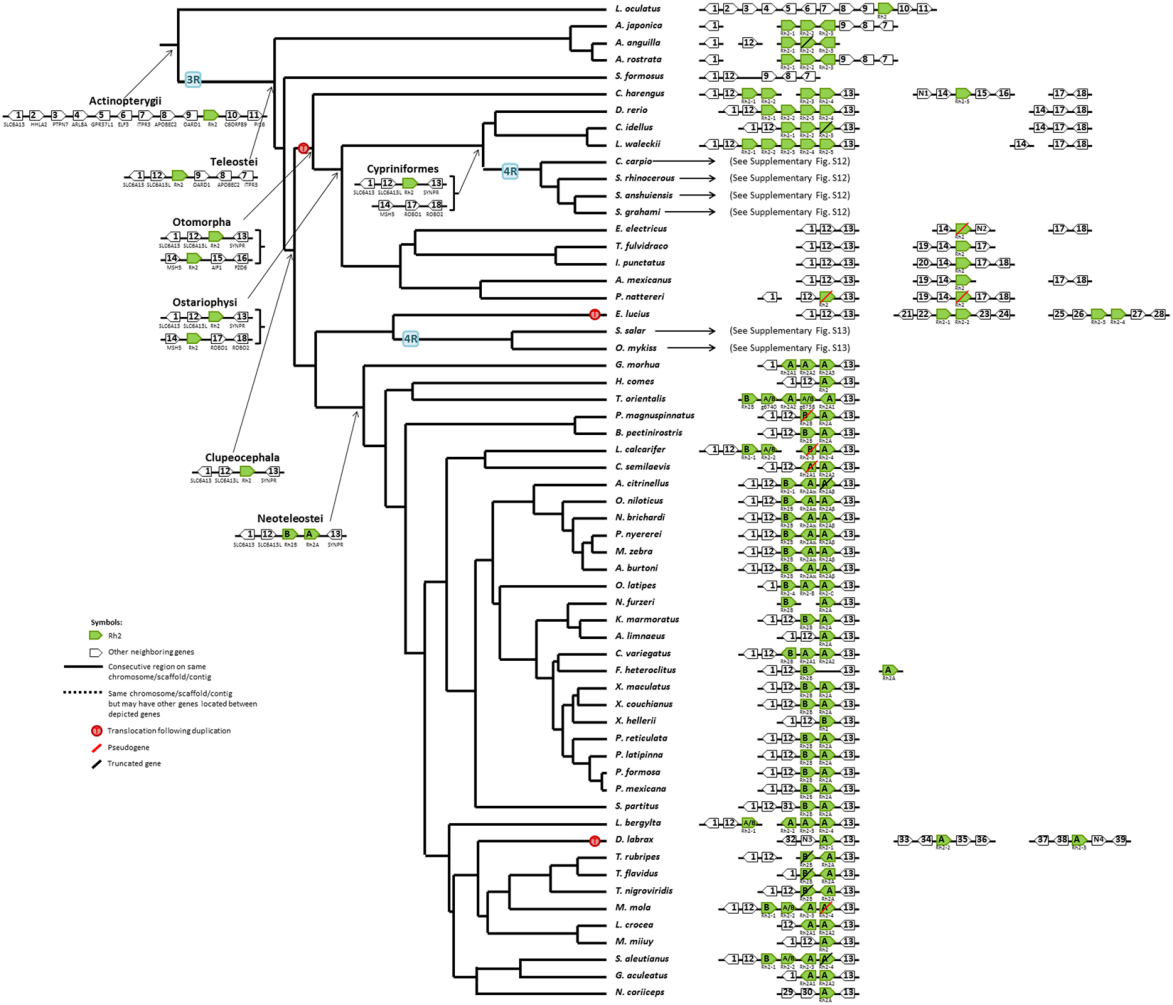
The most parsimonious tree of the Rh2 syntenies. See the legend of Fig. 2.

**Figure 5:**
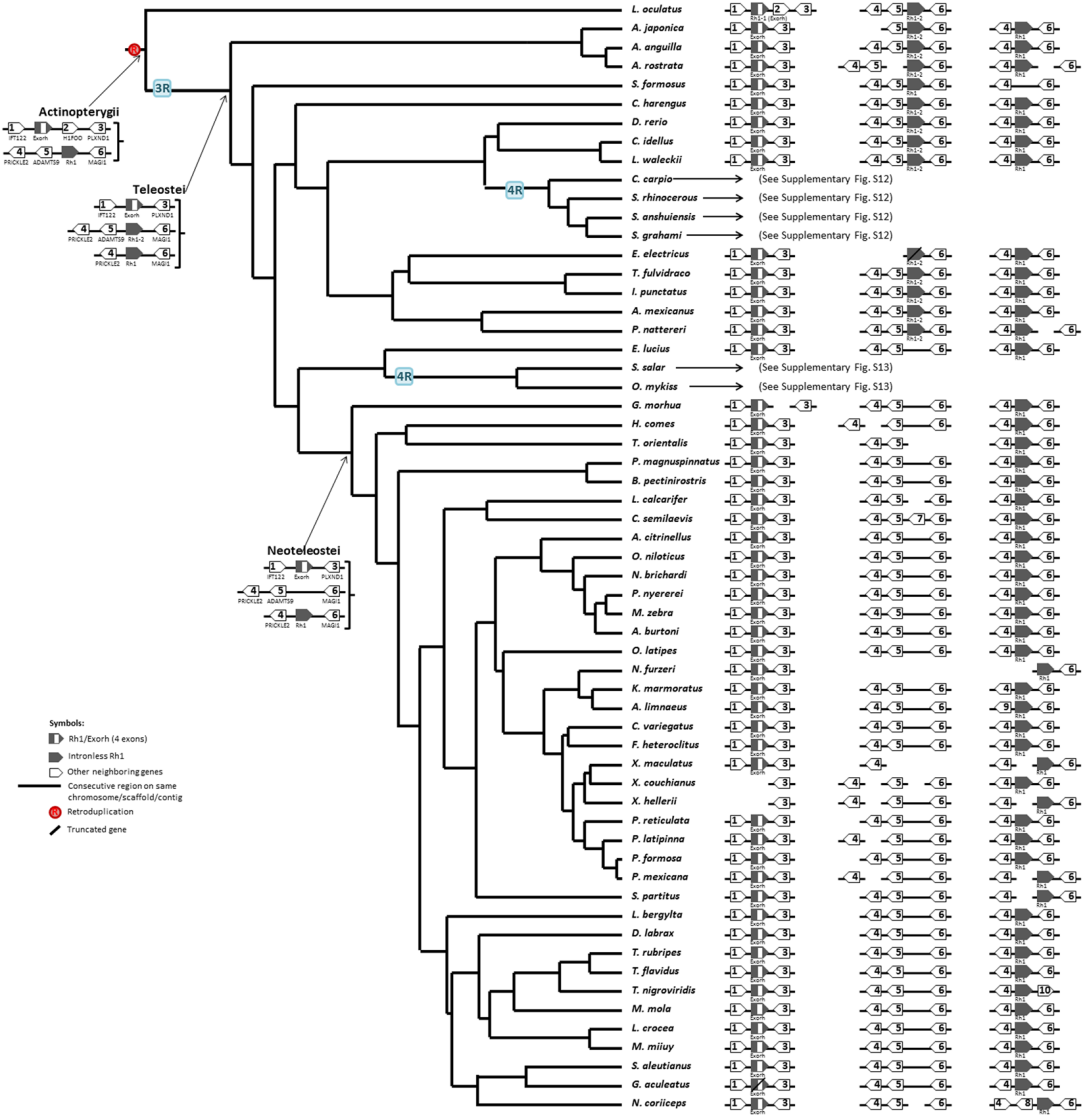
The most parsimonious tree of the Rh1 syntenies. See the legend of Fig. 2.

Here a synteny means the synteny of an opsin gene (or opsin genes) and its adjacent neighboring genes. Our inferences were based on the parsimony principle that requires the minimum number of genomic changes to explain the transition of a gene synteny from one ancestral node to the next (Figs 2–5). For convenience, we used numbers to denote non-opsin genes. However, the numbering system is different in different figures: e.g., Gene1 denotes the HCFC1 gene in Fig. 2, but the TNPO3 gene in Fig. 3 (see also Supplementary Information).

We inferred that the SWS2-LWS synteny in the common ancestor of the Actinopterygii^19^ was Gene1-Gene2-Gene3-Gene4-SWS2-LWS-Gene5-Gene6-Gene7, which is found in the *Lepisosteus oculatus* genome (Fig. 2) and Gene1-Gene2-Gene3-Gene4-SWS2 is observed in coelacanth (data not shown). This synteny lost Gene2 and was duplicated in the 3R event in the common ancestor of Teleostei. One of the two descendant syntenies subsequently lost the opsin genes and became Gene1-Gene3-Gene4-Gene6-Gene7, which will not be considered from now on. The other became Gene1-Gene4-SWS2-LWS-Gene5-Gene7, and then lost Gene4 and became Gene1-SWS2-LWS-Gene5-Gene7 in both the common ancestor of Ostariophysi and the common ancestor of Euteleosteomorpha (Fig. 2). Three translocation events were inferred for LWS. In the *Scleropages formosus* lineage LWS was duplicated twice and one copy was translocated to another chromosome. In the common ancestor of *Astyanax mexicanus* and *Pygocentrus nattereri*, LWS was duplicated and one copy was translocated to another chromosome. In the *Esox lucius* (northern pike) lineage, LWS was tandemly duplicated and then the two LWS genes were duplicated and one duplicate was translocated to another chromosome. A SWS2 tandem duplication occurred in the common ancestor of Neoteleostei and a SWS2A tandem duplication occurred in the ancestor of Percomorpha (Fig. 2). Our inferred pattern of SWS2 duplication is consistent with that of Cortesi *et al*.^26^.

The SWS1 synteny underwent very few changes during fish evolution as in almost all ray-finned fishes the SWS1 genes are located between Gene1 and Gene2 (Fig. 3). The only exception happened in *Austrofundulus limnaeus*, which might be an assembly error because the SWS1 synteny is highly conserved in Cyprinodontiformes (Fig. 3). In addition, we inferred that a SWS1 tandem duplication occurred in *L*. *oculatus* before 3R and another duplication in Cyprinodontiformes after 3R (Fig. 3).

We inferred that the Rh2 synteny in the common ancestor of the Actinopterygii was Gene1-Gene2-Gene3-Gene4-Gene5-Gene6-Gene7-Gene8-Gene9-Rh2-Gene10-Gene11 (Fig. 4). Later, the synteny in the common ancestor of Teleostei became Gene1-Gene12-(Rh2)_n_-Gene9-Gene8-Gene7 because (Rh2)_3_-Gene9-Gene8-Gene7 was found in the eels and Gene1-Gene12-Gene9-Gene8-Gene7 was found in *S*. *formosus*. In the common ancestor of Clupeocephala species, we inferred that the Rh2 synteny became Gene1-Gene12-(Rh2)_n_-Gene13 as it was found in most of the studied Clupeocephala species (Fig. 4). Therefore, the evolution of the Clupeocephala Rh2 synteny from the ancestral Teleostei would require the losses of Gene2, Gene3, Gene4, Gene5, Gene6, Gene10, and Gene11, a duplication and then inversion of Gene1 (named Gene12 in Fig. 4), and translocation of Gene13 from another genomic region (Fig. 4). Then a rearrangement first created the Gene14-Gene15-Gene16 block and a Rh2 duplication and then a translocation occurred in the common ancestor of Otomorpha, resulting in the synteny Gene14-Rh2-Gene15-Gene16 (Fig. 4). Later, a rearrangement changed this synteny block to Gene14-Rh2-Gene17-Gene18 in the common ancestor of Ostarophysi (Fig. 4). The series of changes in this synteny block is supported by the Gene14-Rh2-Gene15-Gene16 block in *Clupea harengus*, the Gene14-Rh2-Gene17-Gene18 block in *Pygocentrus nattereri* and *Ictalurus punctatus*, an incomplete block in *Electrophorus electricus*, *Astyanax mexicanus*, *Pygocentrus nattereri* and *Tachysurus fulvidraco*, and the Gene14-Gene17-Gene18 block with Rh2 lost in Cypriniformes (Fig. 4). Finally, a Rh2 tandem duplication occurred in the common ancestor of Neoteleostei and later diverged into Rh2A and Rh2B (Fig. 4).

Two ancestral-like Rh1 gene syntenies could be found in basal *Lepisosteus oculatus* (Fig. 5). First, Exorh was located in Gene1-Exorh-Gene2-Gene3, this synteny lost Gene2 and after the 3R event in the common ancestor of Teleostei only one copy (Gene1-Exorh-Gene3) was retained. Second, an Rh1 retro-duplication event^3^ generated an intronless Rh1 gene in the synteny Gene4-Gene5-Rh1-Gene6 in *Lepisosteus oculatus*, and this synteny was duplicated in the 3R event but one of the two copies lost Gene5. Therefore, the ancestor of Teleostei possessed the following three syntenies: Gene1-Exorh-Gene3, Gene4-Rh1-Gene6 and Gene4-Gene5-“Rh1-2”-Gene6. Later, the ancestor of Neoteleostei lost the intronless Rh1-2 gene in the Gene4-Gene5-“Rh1-2”-Gene6 block.

Finally, all of the opsin gene syntenies were duplicated in *Cyprinus carpio* and *Sinocyclocheilus spp*., and *C*. *carpio* experienced additional rearrangements (Supplementary Fig. S12). Supplementary Fig. S13 showed that following the 4R event in the ancestor of *Oncorhynchus mykiss* and *Salmo salar*, the duplicated intronless Rh1 and Exorh genes in both species became pseudogenes and a newly duplicated Gene1-Gene12-(Rh2)_4_-Gene13 block lost the Rh2 genes and became Gene1-Gene12-Gene13.

### Living Environment and Opsin Gene Copy Number

We made the following two pair-wise comparisons of fish groups according to their habitats: (1) freshwater vs. marine fishes, and (2) fishes living in water above 30 m vs. fishes living below 50 m (Fig. 1 and Supplementary Table S1). As the freshwater and marine species do not constitute independent samples, we used the phylogenetic comparative analysis^33^. We reached two conclusions (Supplementary Table S5). First, the freshwater fishes possess significantly more SWS1 and LWS genes per species than the marine fishes. Second, fishes living in water above 30 m have significantly more SWS1 and LWS genes but fewer Rh2 genes than fishes living below 50 m (Supplementary Table S5). When we considered the species that only have undergone 3R event, these differences remained significant (Supplementary Table S5).

### Amino Acid Substitutions at Tuning Sites

**Figure 6:**
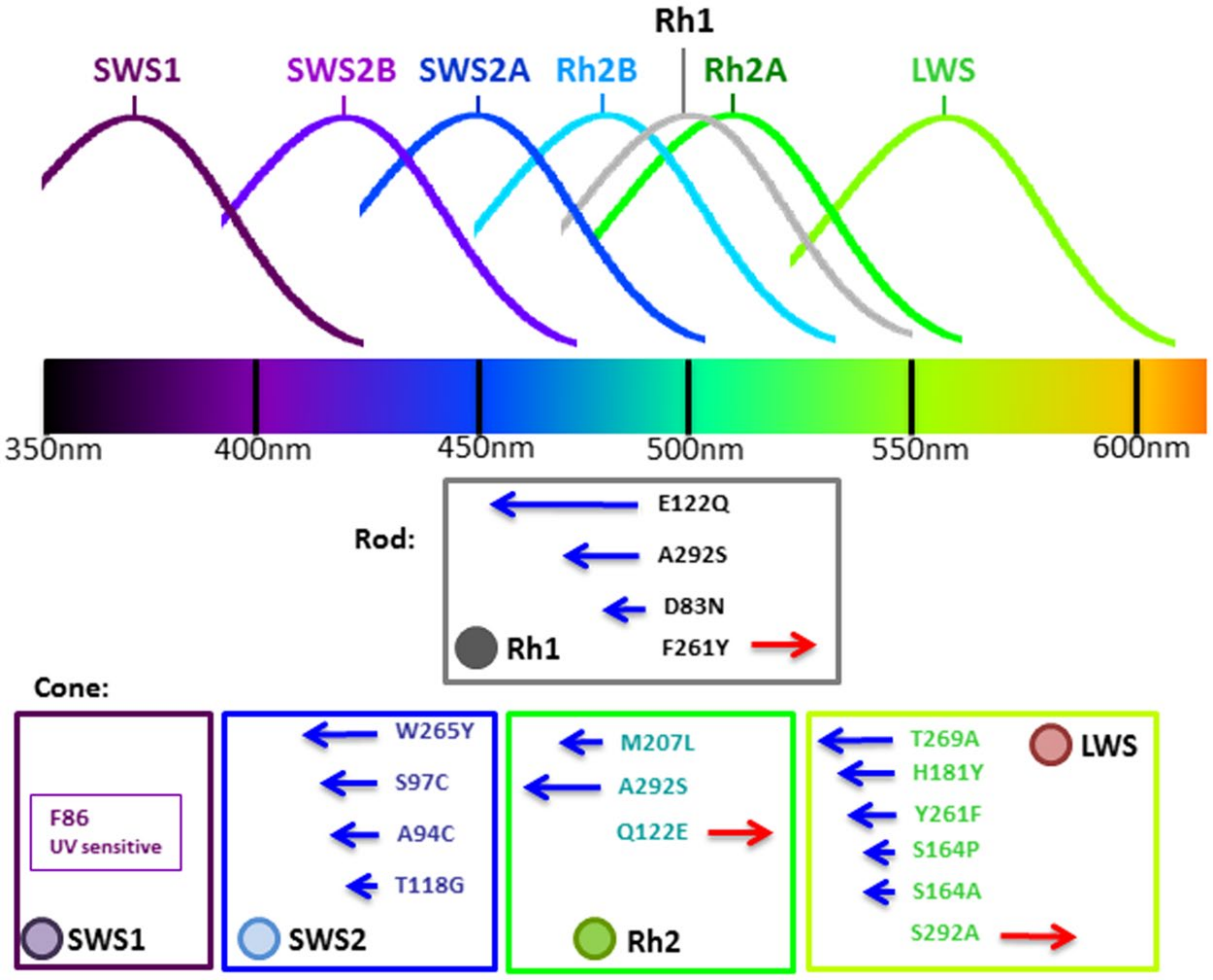
A summary of spectral histograms and key tuning sites of opsins. Changes at tuning sites are denoted by the 1-letter IUPAC amino acid code and residue site numbers. For example, D83N means a change from D to N at residue position 83. The tuning sites are numbered according to bovine rhodopsin.

**Figure 7:**
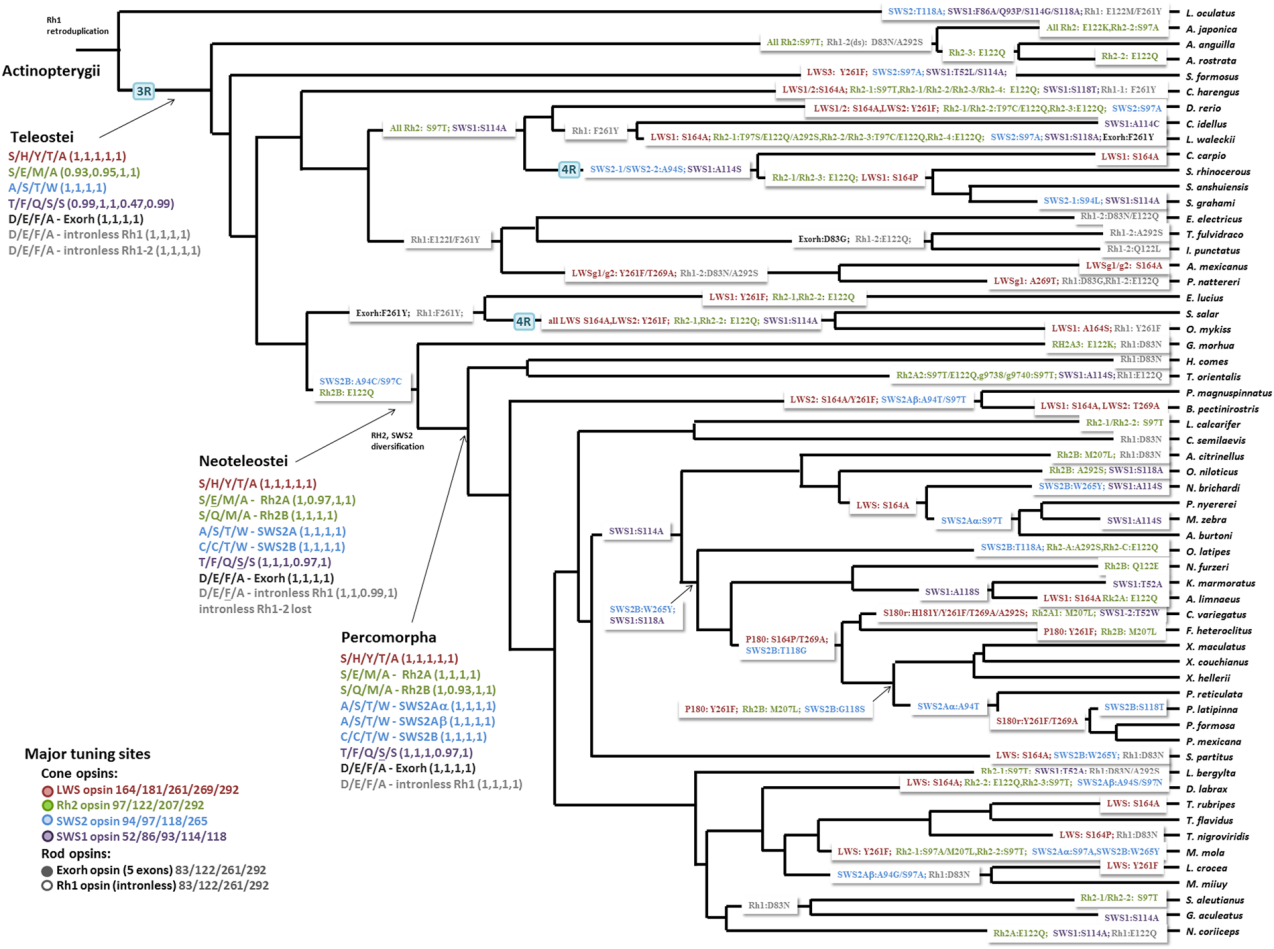
Predicted ancestral amino acid residues at the major tuning sites of opsins. The ancestral amino acid residues of the major tuning sites of opsin genes are shown at the major ancestral nodes (the common ancestor of the 59 fishes, Actinopterygii, Teleostei and Neoteleostei). We use the color code to indicate the opsin family with its member(s) that experienced the described amino acid substitution(s). The amino acid substitutions at tuning sites are indicated on the branches of the species tree. For the Rh2 and SWS2 genes in Neotelestei fishes, we show their subtypes (Rh2A, Rh2B, SWS2A, SWS2B, SWS2Aα and SWS2Aβ). A series of substitutions on the same set of opsins in the same state are separated by “/”, followed by parentheses with the probability of each predicted ancestral amino acid. If not all opsins in the same family of a species/lineage experienced the same set of substitution(s), then we indicate the particular set of opsins experienced the substitution(s). For example, LWS1:S164A on the branch leading to *Cyprinus carpio* indicated that the *C*. *carpio* LWS1 opsin experienced the substitution S164A. Otherwise, a set of substitution(s) without a clear indication of any particular set of opsins means all opsins in the same fish family of a species/lineage shared the same substitution(s). For example, S118A substitution labeled in purple on the common ancestor of *O*. *latipes* and Cyprinodontiformes indicated that the S118A substitution in the SWS1 opsin is present in all of the 13 fishes belonging to this lineage. The tuning sites are numbered according to those in bovine rhodopsin.

To date, more than 30 tuning sites in opsin proteins have been identified to influence the λmax of vertebrate opsins^4,8^. However, many of them have been conserved in Teleostei fishes. For example, C90S and S90C changed the SWS1 λmax significantly in birds, whereas serine is found at this residue site in all of the Teleostei we studied. Therefore, we excluded all such conserved sites. Also we considered only those tuning sites that have been found to have an effect of $\geqq$5 nm spectral shift^4,8,10,32,34,35^. Figure 6 summarizes the changes and their effects with experimental evidence^4,8,10,32,34,35^. We used the species tree (Fig. 1) and maximum likelihood to infer the evolutionary changes at tuning sites (see Materials and Methods). As shown in Fig. 7, in the vast majority of cases the ancestral state of a tuning site was inferred with a probability of 1, and the probability for all other cases with one exception was $\geqq$0.93.

The LWS opsins in this study have exhibited polymorphism across all five key tuning sites (known as “five-sites” rule)^10^. The ancestral amino acids at the five tuning sites in Teleostei LWS were predicted to be S164, H181, Y261, T269, and A292, with the predicted λmax = ~560 nm with a vitamin-A1 chromophore^36^ (Fig. 7). In addition to the substitution events reported in the literature^37^, we identified more substitutions in the LWS phylogenetic tree (Fig. 7). First, S164A, a blue shift, frequently occurred in LWS, i.e., *Stegastes partitus* (bicolor damselfish), *Dicentrarchus labrax*, *Takifugu rubripes*, *Maylandia zebra* (zebra mbuna), *Pundamilia nyererei* (Nyerere’s Victoria Cichlid), *Clupea harengus*, *Oncorhynchus mykiss* and *Salmo salar* (Fig. 7). Second, Y261F, another blue shift, independently occurred in *Mola mola*, *Larimichthus crocea* and *Esox lucius* (Fig. 7). Third, in addition to guppy and killifish^38,39^, S164P also occurred in *Tetraodon nigrovieidis* and the common ancestor of *Sinocyclocheilus spp*. (Fig. 7). Fourth, two substitutions, H181Y and A292S occurred in one duplicated LWS (LWS S180r) of *Cyprinodon variegatus* (sheepshead minnow) (Fig. 2). Fifth, two back-mutations A164S and A269T were observed in *O*. *mykiss* LWS1 and *P*. *nattereri* LWSg3. Finally, multi-substitutions at the five sites, which could induce a larger blue shift, were observed in the lineages with duplication events, i.e., Cyprinodontiformes, Cypriniformes, Salmoniformes, Gobiiformes, Osteoglossiformes and Characiformes.

In SWS2, the ancestral amino acids at the key tuning sites^32^ were predicted to be A94, S97, T118 and W265 (Fig. 7). The tandem duplication of SWS2 in the common ancestor of Neoteleostei fishes led to SWS2A and SWS2B^8^ (Fig. 2), and then A94C and S97C occurred in the ancestor of Neoteleostei SWS2B (Fig. 7). Several amino acid substitutions occurred independently in the SWS2A and SWS2B of Neoteleostei fishes. For example, W265Y occurred in the common ancestor of Cyprinodontiformes and Beloniformes SWS2B, and T118G in SWS2B occurred in the common ancestor of *Fundulus heteroclitus*, *Cyprinodon variegatus*, *Poecilia spp*. and *Xiphophorus spp*. (Fig. 7). On the other hand, substitutions at sites 94 and 97 occurred several times in Percomorpha SWS2A.

For SWS1, F86 and S90 led to the UV sensing of non-avian SWS1 opsin in vertebrates^9^. In this study, F86 and S90 have been conserved in all fishes, except for A86 in *Lepisosteus oculatus*. In addition, substitutions occurred frequently at sites 114 and 118 during teleost SWS1 evolution.

The predicted amino acids of the ancestral Rh2 opsin at the four key tuning sites^35^ are S97, E122, M207 and A292 (Fig. 7). Tandem duplications of Rh2 occurred independently in the common ancestors of many major lineages, i.e., Salmoniformes, Cyprniformes, Anguilliformes, and Neoteleostei. The Rh2 duplication in the common ancestor of Neoteleostei fishes and subsequent amino acid changes led to Rh2A and Rh2B. The blue shift E122Q occurred not only in the common ancestor of Rh2B but also in other lineages, e.g., Salmoniformes and Esociformes. In addition, S97T was observed in the Rh2 of several lineages, i.e., Anguilliformes, Cypriniformes, and some species of Neoteleostei.

There are four tuning sites that can alter Rh1 λmax by >10 nm in fishes^4,8,40^ (Fig. 6), and their ancestral amino acids were predicted to be D83, E122, F261, and A292. (Fig. 7). In the ray-finned fish lineage, Rh1 was retro-duplicated, leading to an intronless Rh1. For the gene with introns, i.e., Exorh, the amino acids at the four tuning sites, D83, E122, F261, and A292, have been conserved in Actinopterygii, except for a few lineages, i.e., Salmoniformes (Fig. 7). In contrast, several substitutions at the tuning sites of the intronless Rh1 occurred. For example, D83N, the most frequent substitution in intronless Rh1, occurred in *Cynoglossus semilaevis* (tongue sole), *Larimichthys crocea* and some other species (Fig. 7). Furthermore, F261Y occurred four times and E122Q occurred twice in Teleostei intronless Rh1 (Fig. 7).

## Discussion

Our opsin gene identification pipeline integrated the information of known opsin genes and genome annotation (Supplementary Fig. S1). It recovered all of the opsin genes identified in two previous studies of nine genomes^21,22^, and also our characterization of SWS2 genes is similar to that of Cortesi *et al*.^26^. However, one caveat is that most of the coding region of Rh2Aβ gene in the *A*. *citrinellus* reference genome was not sequenced, and Torres-Dowdall *et al*.^41^ indeed reported that *A*. *citrinellus* Rh2β is functional.

Synteny analysis facilitates the inference of duplication and translocation events and gene gain and loss events. Our analysis supported the view that all five types of opsins already existed in the common ancestor of ray-finned fishes^42^ (Fig. 1) and revealed that SWS1, Rh1 and Rh2 genes are usually located in different genomic regions, while SWS2 and LWS genes are usually linked together (Figs 2–5, Supplementary Figs S12–S13). These four syntenic regions have evolved by duplication, translocation, and point mutation events^19,43^.

After the 3R event, only the duplicated intronless Rh1 and the SWS2-LWS synteny have been retained in extant teleosts and one of the duplicated SWS2-LWS syntenies lost all LWS and SWS2 genes (Figs 2 and 5). In addition, the evolution of the Rh2 synteny was highly dynamic prior to the emergence of Neoteleostei fishes (Fig. 4). This observation supports the view that the 3R event accelerated the loss of ancestral syntenies^44^. The 4R event in the *Cyprinus carpio* lineage appeared to have been followed by a series of rearrangements, leading to the extant Rh1, Rh2 and Exorh syntenies in carps, while those in *Sinocyclocheilus spp*. underwent no massive rearrangement (Supplementary Fig. S12). On the other hand, after the 4R event in the ancestor of Salmoniformes, a smaller number of duplicated opsin genes were retained (Supplementary Fig. S13).

Our synteny analysis indicated that most gene duplication events involving opsin genes were tandem duplications (Figs 2–5, Supplementary Figs S12 and S13), especially opsins in the LWS and Rh2 families^3^. However, the 4R in the *Cyprinus carpio* and *Sinocyclocheilus spp*. doubled their entire opsin gene repertoire, and that in Salmoniformes contributed an extra copy of the SWS1 gene to *Oncorhynchus mykiss* (Supplementary Figs S12 and S13), which might be helpful for its foraging on zooplankton^45^.

A retro-duplicated Rh1 led to the intronless Rh1 genes in ray-finned fishes^16^ (Figs 1 and 5). Teleosts gained one intronless Rh1 gene. In addition, duplication events gave *Scleropages formosus*, *Clupea harengus*, *Danio rerio*, *Cyprinus carpio*, *Sinocyclocheilus anshuiensis*, *Sinocyclocheilus grahami*, *Astyanax mexicanus*, *Pygocentrus nattereri*, *Esox Lucius*, *Salmo salar* and *Oncorhynchus mykiss* more LWS genes (Fig. 1) and also gave *Anguillia spp*., *C*. *harengus*, *D*. *rerio*, *Ctenopharyngodon idellus*, *Leuciscus waleckii*, *C*. *carpio*, *Sinocyclocheilus spp*., *E*. *Lucius*, *S*. *salar* and *O*. *mykiss* more Rh2 genes. Later, the common ancestor of Neoteleostei lost Rh1-2, while its SWS2 and Rh2 duplicated genes diverged and became SWS2A and SWS2B and Rh2A and Rh2B, respectively (Fig. 1). The most recent common ancestor of Percomorpha gained one SWS2A gene from a tandem duplication (SWS2Aα and SWS2Aβ) (Figs 1 and 2). Zebrafish Rh1-2 shows a blue-shift compared to zebrafish Rh1^46^ and in non-Neoteleostei fishes the Rh1-2 genes are clearly divergent from their Rh1 genes; therefore, it is not clear why Neoteleostei fishes only retain one Rh1 gene^46^. The divergence in Neoteleostei SWS2 and Rh2 duplicated genes conferred better sensing of the blue and the green light spectrum, and the SWS2A in Percomorpha improved their blue-light perception in bluish aqueous habitats^26^.

Freshwater fishes possess more SWS1 and LWS genes than marine fishes (Supplementary Table S5). Most freshwaters are shallow, so that UV light can penetrate through the water. On the other hand, red light is quickly absorbed by water, so that marine fishes in deep water tend to lose LWS genes. Similar results were obtained when we compared the opsin copy number between fishes living above 30 m and those living below 50 m, because shallow water receives more UV and red light than deep water. In addition, phylogenetic comparative analysis also showed marine fishes inhabit below 50 m possess more Rh2 genes than those live above 30 m. Because the blue-green light dominate the photic environment in deep water, the more Rh2 genes a fish possess imply the better visual adaptation in different habitats or/and at different developmental stages, e.g., the pacific blue-fin tuna (Fig. 1). In Neoteleostei fishes, most loss events of Rh2 genes involved Rh2B genes, but the lost part of spectra sensitivity could be covered by SWS2A opsins. *Electrophorus electricus*, *Ictalurus punctatus* and *Tachysurus fulvidraco*, which live in turbid freshwater environments with reduced penetration of short wavelength lights^28,47^, have also lost the SWS1 and SWS2 genes and in *E*. *electricus* the Rh2 gene has become a pseudogene.

Fishes that inhabit diverse water bodies with different spectral backgrounds may have different opsin gene repertoires with tuning site changes that help fine tuning the visual adaptation to their habitats^21,22^ (Figs 6 and 7). For LWS, the LWS phylogeny suggests that amino acid substitutions had occurred in different lineages to adapt to different photic environments (Fig. 7). For example, S164A, a blue shift in LWS, independently occurred in the *Clupea harengus* and *Stegastes partitus* (bicolor damselfish) lineages, enabling them to adapt to deeper ocean (Fig. 7). Substitutions in a duplicated LWS gene may confer a new function. For example, in *Fundulus heteroclitus* (mummichog), *Poecilia reticulata* (guppy) and *Xiphophorus maculatus* (southern platyfish), the spectral sensitivity of the duplicate LWS P180 was shifted to the green light spectrum owing to the three substitutions S164P, Y261F and T269A (Fig. 7). In *Boleophthalmus pectinirostris* (blue-spotted mudskipper), on the other hand, LWS2 had experienced the substitutions S164A, Y261F and S292A, which might have turned LWS2 into a green-light sensitive opsin^22^ (Fig. 7). Therefore, the gene duplication events in LWS and subsequent tuning site changes might have led to a better visual ability to respond to middle-long wavelength lights in some fishes.

In this study, the fishes that possess SWS1 could have UV vision, except for *Lepisosteus oculatus* that has alanine at site 86 (Fig. 7). Site 86 is crucial for UV sensing^10,48^ (Fig. 6), because fishes that lost F86 in SWS1 cannot sense UV-light^48^. The effect of F86A in SWS1 opsin is unknown. However, based on the mutagenesis experiment on *Lepidopus fitchi* SWS1^48^, we hypothesized that F86A might induce a red shiftthat led to a violet sensitive SWS1 opsin (Fig. 6). On the other hand, SWS1 loss events in some fishes could have resulted from environmental adaptation. For example, *P*. *nattereri*, *A*. *mexicanus*, *I*. *punctatus* and *E*. *electricus* live in turbid or tea-stained water, in which UV light could be absorbed very quickly, and these fishes lost the SWS1 genes. Some marine demersal fishes, e.g. *G*. *morhua*, *L*. *crocea*, *M*. *miiuy and C*. *semilaevis*, also lost their SWS1 genes possibly because UV vision is not needed in sandy or muddy habitats. In addition, some vertebrates use color lens or cornea to minimize UV-induced damages, and alter their SWS1 opsin toward violet light sensing^48^. A previous study on mudskipper’s genome hypothesized that the loss of SWS1 might be the consequence of the protection their retinae from UV damage^22^. If this hypothesis holds, the SWS1 loss of shallow water species, such as Tetraodontiformes species and *M*. *mola* that often swim just under sea-surface might have also resulted from the protection against the UV-induced damage.

For SWS2, the λmax in the ancestor of Neoteleostei SWS2A could be 440~460 nm^4^, and A94C and S97C occurred in the ancestor of Neoteleostei SWS2B, shifting its λmax to 400~420 nm^4,34^ (Fig. 7). In SWS2B, two blue shifts, W265Y and T118G, were observed in several lineages. A mutagenesis study showed that A94C and W265Y together in bluefin killifish could result in a 32 nm blue shift, slightly smaller than the sum of the two individual shifts^32^. In SWS2, the interaction between different tuning sites may reduce the effect of the spectral shift induced by individual replacements. Therefore, W265Y and T118G in SWS2B may only create a relatively small spectral shift because of the interaction between tuning sites.

For Rh2, E122Q caused a ~20 nm blue shift in Rh2B in the common ancestor of Neoteleostei; the λmax of Rh2A ranges from 500 to 530 nm, while that of Rh2B from 450 to 500 nm^4^ (Fig. 6). In the Rh2A clade, some substitutions represent the adaptation to extreme light environments. For example, E122Q occurred in *Anguilla spp*. Rh2-3, *Notothenia coriiceps* Rh2A and *Thunnus orientalis* Rh2A2 for adapting to deep sea, deep Arctic and demersal habitats, respectively (Fig. 7). In addition, E122Q and M207L in some Rh2 duplicated genes of *Danio rerio*, *Dicentrarchus labrax*, *Leuciscus waleckii*, *Sinocyclocheilus spp*., *Esox lucius*, *O*. *mykiss*, *S*. *salar*, and M207L in that of *Cyprinodon variegatus*, *Amphilophus citrinellus*, *Mola mola* and several Cyprinodontiformes fishes might have conferred these fishes with different λmax’s for more delicate sensing of blue and green lights (Fig. 7).

In the above we have mentioned some examples of neo-functionization of duplicated genes due to changes at tuning sites. Here we add some possible cases. First, the Rh1 duplication and the D83N/A292S tuning site changes in a duplicated copy led to the two different Rh1 opsins in Anguilliformes, which are utilized in different dwelling habitats at different developmental stages^14^. Second, the E122Q change in Rh2B led to the divergence in λmax between Rh2A and Rh2B in Neoteleostei. Third, A94C/T97C shifted the blue-light sensitive SWS2 opsin to the violet-light sensitive SWS2B opsin^32^. Fourth, H181Y/Y261F/T269A/A292S and S164P/Y261F/T269A led the red-light sensitive LWS opsin to sense green light and divergent functions of LWS duplicates in Cyprinodontiformes^5,49^. In conclusion, opsin gene duplication and tuning site changes may provide fishes with visual diversity to deal with the selection pressure exerted by different environments.

## Materials and Methods

### Data Download

We first collected 76 ray-finned fish genomes from GenBank, RefSeq, other relevant databases and publications^50–55^ (before December 29, 2016). Then we selected 55 assembled genomes that satisfied the criterion N50 $\geqq$ 100,000 bps. In addition, we added *Anguilla anguilla*, *A*. *japonica* and *A*. *rostrata* because they are basal teleosts, and also *Gasterosteus aculeatus* because it is a model organism. Detailed information of the 59 selected ray-finned fish genomes is given in Supplementary Table S1. In addition, we downloaded from GenBank the coding sequences of annotated opsin genes^5,16,26^ (Supplementary Table S3).

### Prediction of Opsin Genes in a Fish Genome

We downloaded the zebrafish genome annotation (Zv10) from NCBI RefSeq, which included the annotation of all zebrafish opsin genes (Supplementary Tables S3). For each of the other fish genomes, the prediction workflow (pipeline) is shown in Supplementary Fig. S1. For a genome with annotation, we used all annotated protein sequences in the genome and BLASTP to search against the zebrafish protein sequences (e-value < 1 × 10^−5^). Only the protein sequences with the best hits to annotated zebrafish opsins were kept (Supplementary Table S3), and the coding sequences of these protein sequences and their genomic locations were retrieved from the genome annotation. To reduce false negatives (i.e., an opsin gene sequence exists but was not recovered by BLASTP) and to handle genomes with no available annotation, the coding sequences and the genomic locations of opsin genes from previous studies^16,26^ (Supplementary Table S3) were used to search against the genome sequences by TBLASTX (e-value < 1 × 10^−5^) and BLASTN (e-value < 1 × 10^−5^), respectively. In addition, we referenced the BAC clones of Watson *et al*.^56^ (accession numbers: GQ999832, GQ999833) to predict the LWS genes in *Xiphophorus hellerii*, because the syntenies in the *X*. *hellerii* genome are incomplete. The coding sequences of putative opsin genes in these genomes were then obtained from the alignment with the best hits. For the cases with N’s (unknown nucleotides) in coding regions, the number of N’s was estimated from the alignment. Finally, we kept the coding sequences corresponding to the genomic regions with BLAST hits in both approaches or just in the direct search against genome sequences if genome annotation was not available (Fig. S1). A gene is defined as a pseudogene if a premature stop-codon(s) is found in its coding region. A gene is truncated if any of its exons are missing or the number of N’s in its coding region is >100. The genomic information of all identified opsin coding sequences is summarized in Supplementary Table S2.

### Synteny Analysis

To examine the synteny of opsin genes, we checked the genes that have been reported previously in the immediate upstream or downstream neighboring regions of an opsin gene^21,22,26^. These genes are called “typical adjacent genes” here. The locations of these genes in the studied genomes were obtained from genome annotation or using BLAST (e-value < 1 × 10^−5^). The gene order and orientation in the syntenic region were defined based on the original genome annotation. We used a sliding window approach to check whether any typical adjacent genes appeared in nearby regions. The size of the sliding window was set to be 6 + n, where n is the number of opsin genes in the genomic region (i.e., n opsin genes plus 3 upstream and 3 downstream genes). If none of the typical adjacent genes could be found, we reported the three genes upstream and three genes downstream of the opsin genes. There were two cases where manual adjustments were required. The first case was the Gene1-Gene2-Gene3-Gene4-SWS2-LWS-Gene5-Gene6-Gene7 (HCFC1-TMEM187-IRAK1-MECP2-SWS2-LWS-GNL3L-FGD1-TFE3) synteny in *L*. *oculatus*, because there were three genes between HCFC1 and SWS2 (Fig. 2). The other case was the Rh2 synteny in *L*. *oculatus* because there were 8 genes (Gene2~Gene9) between Gene1 (SLC6A13) and Rh2 (Fig. 5). For those genomes without annotation, the sequences of upstream or downstream genes in closely related species that have been annotated were used to search the location of each of these genes by BLASTN and TBLASTN (e-value < 1 × 10^−5^). In addition, we referenced the BAC clones of Watson *et al*.^56^ (accession numbers: GQ999832 and GQ999833) and predicted the following two syntenies that are related to LWS and SWS2 in *X*. *hellerii* HCFC1 - SWS2Aa - SWS2B - LWS S180-1 - LWS P180 - LWS S180-2 - GNL3L - TFE3 and LWS S180r, which is located in the intron of the gephyrin (GPHN) gene. These two inferred syntenies are exactly the same as those annotated by Watson *et al*.^56^.

We inferred the minimum number of synteny changes in each branch by the parsimony principle. We assumed that a translocation event happened if an opsin gene appeared in a new synteny that was different from the synteny at the immediate ancestral node (Figs 2–5). The inferred syntenies at each evolutionary node are summarized in Figs 2–5, Supplementary Table S4 and Figs S12–S13.

### Construction of Phylogenetic Trees of Opsin Genes

To have a good reference for predicting the duplication and diversification pattern of opsin genes, we used the predicted opsin gene sequences for each opsin families. The sequences which were regarded as pseudogenes and truncated genes were excluded in the construction of phylogenetic trees. The selected gene sequences of each families were first aligned by CLUSTALW^57^. The phylogenetic trees was inferred by using the Maximum Likelihood method based on the General Time Reversible model considering a discrete Gamma distribution for modeling evolutionary rate differences among sites (5 categories) and meanwhile allowed some sites to be evolutionarily invariable (GTR + G + I). The bootstrap procedure was repeated for 500 times. The entire process was done using MEGA 6.0 software^58^. The phylogenetic trees are shown in Supplementary Figs S2–S6.

### Inference of Copy Number Changes

As mentioned in Results, in inferring copy number changes, we used the parsimony principle to infer the minimum number of copy number changes in each branch of Fig. 1; see Supplementary Figs S7–S11 for the copy number changes in each opsin gene family. Then we checked if the minimum number of changes in a branch was consistent with the inferred synteny changes in that branch. We assumed that a WGD event (3R or 4R) doubled the copy number of each existing opsin gene. Also some tandem duplication events can be inferred from synteny changes. For example, for the SWS2 family, the tandem duplication event that produced SWS2A and SWS2B in the latest common ancestor of Neoteleostei fishes is supported by the predicted synteny Gene1-SWS2A-SWS2B-LWS-Gene5-Gene7 in the Neoteleostei fishes and the duplication event that produced SWS2Aα and SWS2Aβ in the latest common ancestor of Percomorpha fishes was supported by the predicted synteny Gene1-SWS2Aβ-SWS2Aα-SWS2B-LWS-Gene5-Gene7 in the Percomorpha fishes (Fig. 2). For the Rh2 family, the duplication event that produced Rh2A and Rh2B in the latest common ancestor of Neoteleostei was supported by the predicted Gene1-Gene12-Rh2B-Rh2A-Gene13 synteny in Neoteleostei fishes (Fig. 4). For the Rh1 family, the loss of Rh1-2 in the latest common ancestor of Neoteleostei was supported by the fact that all the predicted Rh1-2 syntenies in Neoteleostei fishes lost the Rh1-2 gene (Fig. 5).

### Ancestral Sequence Prediction

We only considered complete opsin genes since truncated genes and pseudogenes are unlikely to be functional. In general, we selected the gene that is most similar to the counterpart in *L*. *oculatus* as the representative for each opsin gene family. The representative gene sequences were then aligned by CLUSTALW^57^ for codon-based alignment. The species tree (Fig. 1) was used as the reference tree when using maximum likelihood approach to infer the ancestral states. The predicted syntenies (Figs 2–5) were also employed to help assign the homology between genes and determine the ancestral state of the tuning site. The analysis was done by using MEGA 6.0 software^58^.

### Statistical tests

Using Fishbase (Supplementary Table S1), we classified the studied fishes into two pair-wise comparisons: (1) freshwater vs. marine fishes, and (2) living water depth: <30 m vs. >50 m (Fig. 1).

To take the fish phylogeny into consideration for statistical testing, we conducted the Phylogenetic Comparative Analysis^33^. The cladogram of the 59 ray-finned fishes (Fig. 1) was used as the reference phylogeny. The species divergence times were obtained from the literature we referenced when constructing Fig. 1 ^23,29,30^, the divergence time estimated by TimeTree^30^ was taken when there were conflicts between different studies. For the internal nodes with unknown divergence time, we randomly assigned values that are not contradicting to the other constraints. For each test, four models of opsin gene copy number evolution were formulated. The first model (denoted as BM) is based on Brownian motion, which assumes that the evolution is a result of pure drift. It served as the null hypothesis. The rest three models which served as alternative hypotheses are based on Ornstein‐Uhlenbeck process, which takes both drift and selection into consideration. The second model (denoted as OU(1)) assumed a global optimum for opsin gene copy number. The third model (denoted as OU(2)) assumed two different global optimum for opsin gene copy number as there are two types of habitats in each test. For OU(2), the habitats of ancestral states were inferred based on maximum parsimony and bottom-up principle. The last model (denoted as OU(3)), which is an extension of OU(2), assumed that the habitat in ancestral state is unknown. The parameters of each model were estimated by likelihood maximization. Finally, the likelihood ratio test, Akaike Information Criterion and Schwarz Information Criterion were used to assess which model fits better to your data. For likelihood ratio test, we regarded a test as statistically significant if the p-value is $\leqq$0.05. For cases with either OU(2) and OU(3) as significant, we regarded them as cases number of opsin genes differs significantly between the two groups. Details of phylogenetic comparative analysis are summarized in Supplementary Table S5.

## Electronic supplementary material

Supplementary Information

Dataset 1

Dataset 2

Dataset 3

Dataset 4

Dataset 5

## References

[1] Terakita A (2005). The opsins. Genome Biol.

[2] Kefalov VJ, Cornwall MC, Fain GL (2010). Physiological studies of the interaction between opsin and chromophore in rod and cone visual pigments. Methods Mol Biol.

[3] Fitzgibbon J (1995). The rhodopsin-encoding gene of bony fish lacks introns. Gene.

[4] Bowmaker JK (2008). Evolution of vertebrate visual pigments. Vision Res.

[5] Davies WL, Collin SP, Hunt DM (2009). Adaptive gene loss reflects differences in the visual ecology of basal vertebrates. Mol Biol Evol.

[6] Nathans J (1990). Determinants of visual pigment absorbance: role of charged amino acids in the putative transmembrane segments. Biochemistry.

[7] Takahashi Y, Ebrey TG (2003). Molecular basis of spectral tuning in the newt short wavelength sensitive visual pigment. Biochemistry.

[8] Yokoyama S (2008). Evolution of dim-light and color vision pigments. Annu. Rev. Genomics Hum. Genet..

[9] Hunt DM (2007). Spectral tuning of shortwave‐sensitive visual pigments in vertebrates. Photochemistry and Photobiology.

[10] Yokoyama S, Radlwimmer FB (1998). The “five-sites” rule and the evolution of red and green color vision in mammals. Mol Biol Evol.

[11] Yokoyama S, Tada T (2003). The spectral tuning in the short wavelength-sensitive type 2 pigments. Gene.

[12] Varela AI, Ritchie PA (2015). Critical amino acid replacements in the rhodopsin gene of 19 teleost species occupying different light environments from shallow-waters to the deep-sea. Environmental Biology of Fishes.

[13] Jerlov, N. G. & Nielsen, E. S. *Optical aspects of oceanography*. **12** (Academic Press London, 1974).

[14] Wang FY, Fu WC, Wang IL, Yan HY, Wang TY (2014). The giant mottled eel, Anguilla marmorata, uses blue-shifted rod photoreceptors during upstream migration. PLoS One.

[15] Laver CR, Taylor JS (2011). RT-qPCR reveals opsin gene upregulation associated with age and sex in guppies (*Poecilia reticulata*)-a species with color-based sexual selection and 11 visual-opsin genes. BMC evolutionary biology.

[16] Rennison DJ, Owens GL, Taylor JS (2012). Opsin gene duplication and divergence in ray-finned fish. Mol Phylogenet Evol.

[17] Kuraku S, Meyer A, Kuratani S (2009). Timing of genome duplications relative to the origin of the vertebrates: did cyclostomes diverge before or after?. Mol Biol Evol.

[18] Macqueen DJ, Johnston IA (2014). A well-constrained estimate for the timing of the salmonid whole genome duplication reveals major decoupling from species diversification. Proc Biol Sci.

[19] Lagman D (2013). The vertebrate ancestral repertoire of visual opsins, transducin alpha subunits and oxytocin/vasopressin receptors was established by duplication of their shared genomic region in the two rounds of early vertebrate genome duplications. BMC Evol Biol.

[20] Li JT (2015). The fate of recent duplicated genes following a fourth-round whole genome duplication in a tetraploid fish, common carp (Cyprinus carpio). Sci Rep.

[21] Nakamura Y (2013). Evolutionary changes of multiple visual pigment genes in the complete genome of Pacific bluefin tuna. Proceedings of the National Academy of Sciences of the United States of America.

[22] You X (2014). Mudskipper genomes provide insights into the terrestrial adaptation of amphibious fishes. Nature communications.

[23] Brawand D (2014). The genomic substrate for adaptive radiation in African cichlid fish. Nature.

[24] Spady, T. C. *Cichlids as a Model for the Evolution of Visual Sensitivity*. (University of New Hampshire (Department of Zoology), 2006).

[25] Spady TC (2005). Adaptive molecular evolution in the opsin genes of rapidly speciating cichlid species. Mol Biol Evol.

[26] Cortesi F (2015). Ancestral duplications and highly dynamic opsin gene evolution in percomorph fishes. Proc Natl Acad Sci USA.

[27] Koepfli KP, Paten B, Genome KCOS, O’Brien SJ (2015). The Genome 10K Project: a way forward. Annu Rev Anim Biosci.

[28] Froese, R. & Pauly, D. *FishBase*. *World Wide Web Electronic Publication*. http://www.fishbase.org/ (2015).

[29] Betancur-R R (2017). Phylogenetic classification of bony fishes. BMC evolutionary biology.

[30] Kumar S, Stecher G, Suleski M, Hedges SB (2017). TimeTree: a resource for timelines, timetrees, and divergence times. Molecular Biology and Evolution.

[31] Reznick DN, Furness AI, Meredith RW, Springer MS (2017). The origin and biogeographic diversification of fishes in the family Poeciliidae. PloS one.

[32] Yokoyama S, Takenaka N, Blow N (2007). A novel spectral tuning in the short wavelength-sensitive (SWS1 and SWS2) pigments of bluefin killifish (Lucania goodei). Gene.

[33] Cressler CE, Butler MA, King AA (2015). Detecting adaptive evolution in phylogenetic comparative analysis using the Ornstein–Uhlenbeck model. Systematic biology.

[34] Shi Y, Radlwimmer FB, Yokoyama S (2001). Molecular genetics and the evolution of ultraviolet vision in vertebrates. Proceedings of the National Academy of Sciences.

[35] Takenaka N, Yokoyama S (2007). Mechanisms of spectral tuning in the RH2 pigments of Tokay gecko and American chameleon. Gene.

[36] Yokoyama S, Radlwimmer FB (2001). The molecular genetics and evolution of red and green color vision in vertebrates. Genetics.

[37] Yokoyama S, Yang H, Starmer WT (2008). Molecular basis of spectral tuning in the red- and green-sensitive (M/LWS) pigments in vertebrates. Genetics.

[38] Ward MN (2008). The molecular basis of color vision in colorful fish: four long wave-sensitive (LWS) opsins in guppies (Poecilia reticulata) are defined by amino acid substitutions at key functional sites. BMC Evol Biol.

[39] Watson CT (2011). Gene duplication and divergence of long wavelength-sensitive opsin genes in the guppy, Poecilia reticulata. J Mol Evol.

[40] Yokoyama S, Tada T, Zhang H, Britt L (2008). Elucidation of phenotypic adaptations: Molecular analyses of dim-light vision proteins in vertebrates. Proceedings of the National Academy of Sciences of the United States of America.

[41] Torres-Dowdall, J. *et al*. Rapid and parallel adaptive evolution of the visual system of Neotropical Midas cichlid fishes. *Molecular biology and evolution* (2017). 10.1093/molbev/msx143 28444297

[42] Yokoyama S, Yokoyama R (1996). Adaptive evolution of photoreceptors and visual pigments in vertebrates. Annual Review of Ecology and Systematics.

[43] Lamb TD (2016). Evolution of Vertebrate Phototransduction: Cascade Activation. Molecular biology and evolution.

[44] Amores A, Catchen J, Ferrara A, Fontenot Q, Postlethwait JH (2011). Genome evolution and meiotic maps by massively parallel DNA sequencing: spotted gar, an outgroup for the teleost genome duplication. Genetics.

[45] Novales Flamarique I (2013). Opsin switch reveals function of the ultraviolet cone in fish foraging. Proc Biol Sci.

[46] Morrow JM (2017). A second visual rhodopsin gene, rh1-2, is expressed in zebrafish photoreceptors and found in other ray-finned fishes. J Exp Biol.

[47] Enright, J. M. *et al*. Cyp27c1 Red-Shifts the Spectral Sensitivity of Photoreceptors by Converting Vitamin A into A. *Curr Biol*, doi:10.1016/j.cub.2015.10.018 (2015). 10.1016/j.cub.2015.10.018 PMC4910640 26549260

[48] Tada T, Altun A, Yokoyama S (2009). Evolutionary replacement of UV vision by violet vision in fish. Proceedings of the National Academy of Sciences of the United States of America.

[49] Davies WL (2009). Into the blue: gene duplication and loss underlie color vision adaptations in a deep-sea chimaera, the elephant shark Callorhinchus milii. Genome Res.

[50] Benson DA (2013). GenBank. Nucleic Acids Res.

[51] Gallant JR (2014). Nonhuman genetics. Genomic basis for the convergent evolution of electric organs. Science.

[52] Pruitt KD (2014). RefSeq: an update on mammalian reference sequences. Nucleic Acids Res.

[53] Tang H (2015). ALLMAPS: robust scaffold ordering based on multiple maps. Genome biology.

[54] Tine M (2014). European sea bass genome and its variation provide insights into adaptation to euryhalinity and speciation. Nat Commun.

[55] Wang Y (2015). The draft genome of the grass carp (Ctenopharyngodon idellus) provides insights into its evolution and vegetarian adaptation. Nat Genet.

[56] Watson CT, Lubieniecki KP, Loew E, Davidson WS, Breden F (2010). Genomic organization of duplicated short wave-sensitive and long wave-sensitive opsin genes in the green swordtail, Xiphophorus helleri. BMC evolutionary biology.

[57] Thompson, J. D., Gibson, T. & Higgins, D. G. Multiple sequence alignment using ClustalW and ClustalX. *Current protocols in bioinformatics*, 2.3. 1–2.3. 22 (2002). 10.1002/0471250953.bi0203s00 18792934

[58] Tamura K, Stecher G, Peterson D, Filipski A, Kumar S (2013). MEGA6: Molecular Evolutionary Genetics Analysis version 6.0. Molecular biology and evolution.

