# Supplementary material for: The rises and falls of opsin genes in 59 ray-finned fish genomes and their implications for environmental adaptation
Source: Sci Rep. 2017 Nov 14;7:15568. doi: 10.1038/s41598-017-15868-7 (PMC5686071; doi:10.1038/s41598-017-15868-7)
Supplement: Supplementary file 1 — Supplementary Information [file 41598_2017_15868_MOESM1_ESM.pdf]

The rises and falls of opsin genes in 59 ray-finned fish genomes and their implications for environmental adaptation

Jinn-Jy Lin[1,2,3,+], Feng-Yu Wang[4,+], Wen-Hsiung Li[1,3,5*] and Tzi-Yuan Wang[3,*]

[1]Institute of Molecular and Cellular Biology, National Tsing Hua University, Hsinchu 30013, Taiwan
[2]Bioinformatics Program, Taiwan International Graduate Program, Institute of Information Science, Academia Sinica, Nankang, Taipei 11529, Taiwan
[3]Biodiversity Research Center, Academia Sinica, Nankang, Taipei 11529, Taiwan
[4]Taiwan Ocean Research Institute, National Applied Research Laboratories, Kaohsiung 852, Taiwan
[5] Department of Ecology and Evolution, University of Chicago, Chicago 60637, USA
[*]Corresponding authors: whli@sinica.edu.tw, tziyuan@gmail.com
[+]these authors contributed equally to this work

Supplementary information

Complete list of neighboring genes in Figure 2-5

Complete list of neighboring genes in Fig. 2 (identified syntenies of LWS and SWS2 genes in fish genomes): We labeled the neighboring genes of LWS or/and SWS2 with known names or function by the numbers 1~25: 1. HCFC1 (host cell factor C1); 2. TMEM187 (transmembrane protein 187); 3. IRAK1 (interleukin-1 receptor-associated kinase 1); 4. MECP2 (methyl CpG binding protein 2); 5. GNL3L (guanine nucleotide binding protein-like 3-like); 6. FGD1 (FYVE, RhoGEF and PH domain containing 1); 7. TFE3 (transcription factor binding to IGHM enhancer 3); 8. C2 (complement C2); 9. PKNP (polynucleotide kinase 3'-phosphatase); 10. PHACTR3B (phosphatase and actin regulator 3b); 11. RAB7 (ras-related protein Rab-7); 12. TAGLN (transgelin); 13. TPPP3 (tubulin polymerization-promoting protein family member 3); 14. TRIM16 (tripartite motif-containing protein 16); 15. NFKBIB (NF-kappa-B inhibitor beta); 16. EIF4G1 (eukaryotic translation initiation factor gamma 1); 17.

TBC1D15 (TBC1 domain family, member 15); 18. TRHDE (thyrotropin-releasing hormone degrading enzyme); 19. IRAK3 (interleukin-1 receptor-associated kinase 3); 20. HELB (helicase B); 21. UBAP2 (ubiquitin associated protein 2); 22. DIDO1 (death-inducer obliterator 1); 23. GPHN (gephyrin); 24. FMNL14 (formin-like protein 14); and 25. GATA1A (GATA binding protein 1a).

For the neighboring genes whose function are unknown, we labeled them with the numbers N1~N11 and the 1-1 correspondence between gene ID in genome annotation (see Supplementary Table S1 for the source of gene annotation) and the serial number are as follows: N1. NCBI gene ID 105898975; N2. NCBI gene ID 564674; N3. NCBI gene ID 108440552; N4. NCBI gene ID 103384378; N5. NCBI gene ID 102300940; N6. NCBI gene ID 101477056; N7. NCBI gene ID 102201190; N8. NCBI gene ID 105919251; N9. NCBI gene ID 103460226; N10. NCBI gene ID 106936404 and N11. NCBI gene ID 104935480.

Complete list of neighboring genes in Fig. 3 (identified SWS1 gene syntenies in fish genomes): We labeled neighboring genes with known names or function with the numbers 1~8: 1. TNPO3 (transportin 3); 2. CALU (calumenin); 3. IMPG2 (interphotoreceptor matrix proteoglycan 2); 4. ABI3BP (ABI family member 3 binding protein); 5. ADGRG7 (adhesion G protein-coupled receptor G7); 6. SCO2 (SCO2, cytochrome c oxidase assembly protein); 7. NCAPH2 (non-SMC condensin II complex subunit H2) and 8. PTPRO (protein tyrosine phosphatase, receptor type O).

Complete list of neighboring genes in Fig. 4 (identified Rh2 gene syntenies in fish genomes): We labeled neighboring genes with known names or function with the numbers 1~37: 1. SLC6A13 (solute carrier family 6 (neurotransmitter transporter), member 13); 2. HHLA2 (HERV-H LTR-associating protein 2); 3. PTPN7 (tyrosine-protein phosphatase non-receptor type 7); 4. ARL8A (ADP-ribosylation factor-like 8A); 5. GPR37L1 (prosaposin receptor GPR37L1); 6. ELF3 (ETS-related transcription factor Elf-3); 7. ITPR3 (inositol 1,4,5-trisphosphate receptor, type 3); 8. APOBEC2 (apolipoprotein B mRNA editing enzyme, catalytic polypeptide-like 2); 9. OARD1 (O-acyl-ADP-ribose deacylase 1); 10. C6ORF89 (chromosome 6 open reading frame 89); 11. PI16 (peptidase inhibitor 16); 12. SLC6A13L (solute carrier family 6 (neurotransmitter transporter), member 13-like); 13. SYNPR (synaptoporin); 14. MSH5 (mutS homolog 5); 15. AIF1 (allograft inflammatory factor 1); 16. FZD6 (frizzled class receptor 6); 17. ROBO1 (roundabout guidance receptor 1); 18. ROBO2 (undabout guidance receptor 2); 19. SPACA4 (sperm acrosome membrane-associated protein 4); 20. KIFAP3 (kinesin-associated protein 3); 21. SLC2A11 (solute carrier family 2 (facilitated glucose transporter), member 11); 22. TGFA (transforming growth factor, alpha); 23. HDHD3 (haloacid dehalogenase-like hydrolase domain containing 3); 24. TRUB2 (TruB pseudouridine (psi) synthase family member 2); 25. ASCL5 (achaete-scute family bHLH transcription factor 5); 26. CACNA1S (calcium channel,

voltage-dependent, L type, alpha 1S subunit); 27. KCTD20 (potassium channel tetramerization domain containing 20); 28. STK38 (serine/threonine-protein kinase 38-like); 29. POMGNT2 (protein O-linked mannose N-acetylglucosaminyltransferase 2 (beta 1,4-)); 30. SNFK (SNF related kinase); 31. CCDC151 (coiled-coil domain-containing protein 151); 32. H1FOO (H1 histone family, member O, oocyte-specific); 33. TX1 (transposon tx1 uncharacterized 149 kda); 34. BTNL10 (butyrophilin-like protein 10); 35. LRRC57 (leucine-rich repeat-containing protein 57); 36. CaSRL (extracellular calcium-sensing receptor-like); 37. TNNT1 (troponin slow skeletal muscle-like); 38. MOG (myelin-oligodendrocyte glycol); and 39. OR11A1 (olfactory receptor 11a1-like).

For the neighboring genes whose function is unknown, we labeled them with the numbers N1~N4 and the 1-1 correspondence between gene ID in genome annotation (see Supplementary Table S1 for the source of gene annotation) and the serial number is as follows: N1. NCBI gene ID 105890428; N2. *E. electricus* gene ID scaffold7234.g4; N3. *D. labrax* gene ID DLAgn_00093850; and N4. *D. labrax* gene ID DLAgn_00257800.

Complete list of neighboring genes in Fig. 5 (identified Rh1 gene syntenies in fish genomes): We labeled neighboring genes of Rh1 gene(s) with known names or function with the numbers 1~10: 1. IFT122 (intraflagellar transport 122); 2. H1FOO (H1 histone family, member O, oocyte-specific); 3. PLXND1 (plexin D1); 4. PRICKLE2 (prickle homolog 2); 5. ADAMTS9 (ADAM metallopeptidase with thrombospondin type 1 motif, 9); 6. MAGI1 (membrane associated guanylate kinase, WW and PDZ domain containing 1); 7. C2orf16 (uncharacterized protein C2orf16-like); 8. PPHLN1 (periphilin-1); 9. LBX1 (ladybird homeobox homolog 1) and 10. REN (renin).

Supplementary Tables

Supplementary Table S1. List of the fish species included in this study. The list includes the taxonomic and phylogenetic information. The classification of ray-finned fishes was adapted from FishBase and related studies. The information of living environments, habitats and migratory behavior was obtained from FishBase. The sources of genome assembly and annotation were also indicated.

Supplementary Table S2. The coding sequences of all opsin genes identified in this study. For each identified opsin gene, we indicate its name, associated species, length, sequence completeness (complete, truncated, or pseudogene), the tuning sites of the encoded opsin and the coding sequences.

Supplementary Table S3. The coding sequences of all opsin genes obtained from previous studies. For each opsin gene, we indicate its NCBI Genbank accession number, the PubMed ID of the associated study and the coding sequence.

Supplementary Table S4. The genomic locations of the opsin gene syntenies identified in the ray-finned fish genomes. For each identified synteny, we indicate its genomic location and the genes on the synteny.

Supplementary Table S5. The phylogenetic comparative analysis for opsin gene number differences between two groups. The tests which are statistically significant are labeled in red. .

(Supplementary Tables S1-S5 are available as single .xlsx files)

Supplementary Figures

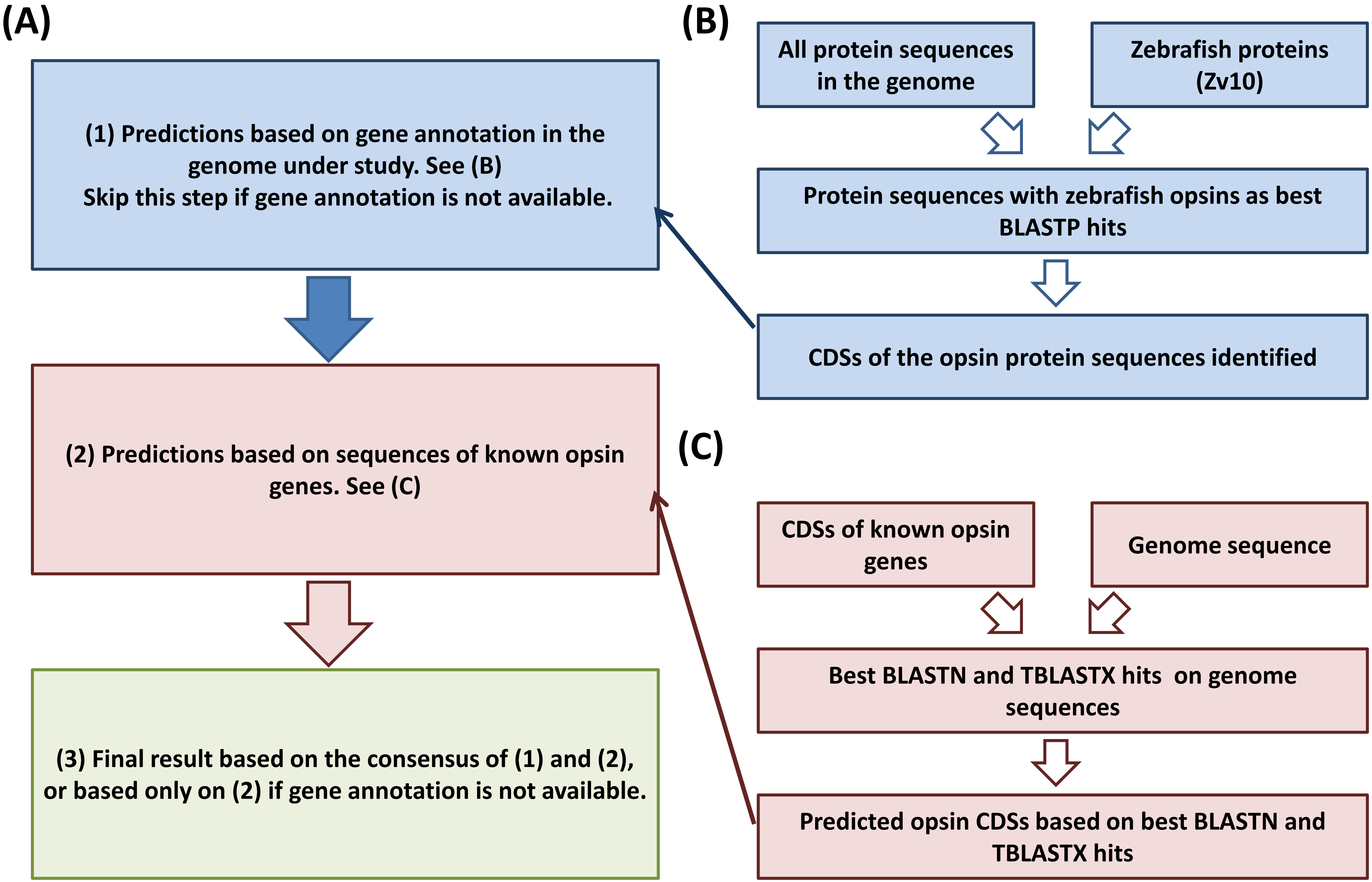

Supplementary Figure S1 Bioinformatics pipeline for identifying opsin genes in fish genomes. (A) The whole pipeline. (B) The detailed procedure of step 1. (C) The detailed procedure of step 2.

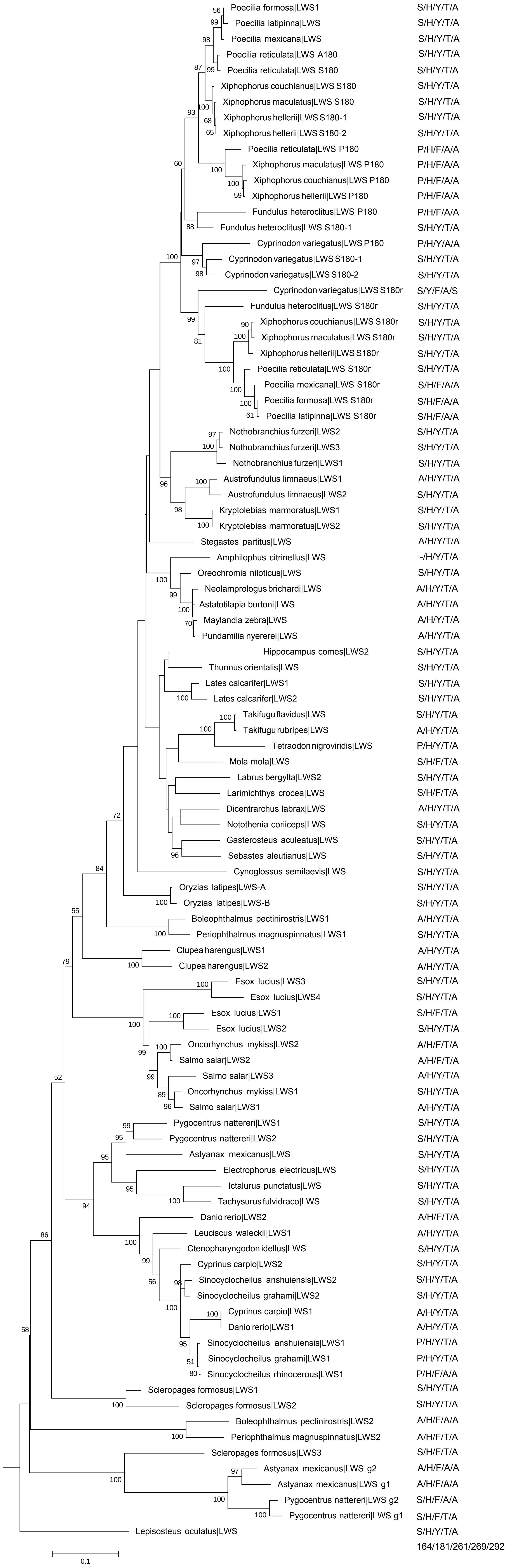

Supplementary Figure S2 Maximum likelihood phylogeny of the complete LWS genes. For each complete LWS gene, we also indicate its key tuning sites (164/181/261/269/292). A tuning site is labeled by "-" if its corresponding coding region was not sequenced.

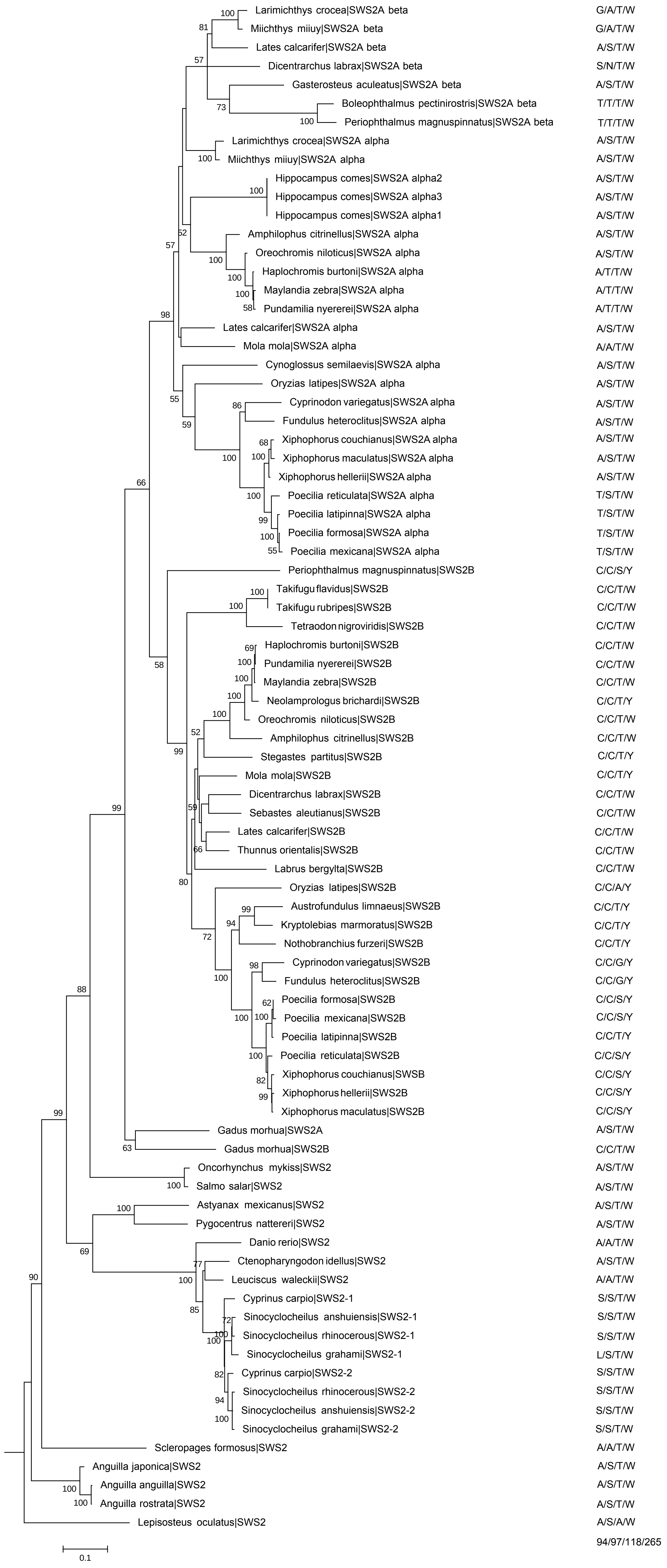

Supplementary Figure S3 Maximum likelihood phylogeny of the complete SWS2 genes. For each complete LWS gene, we also indicate its key tuning sites (94/97/118/265).

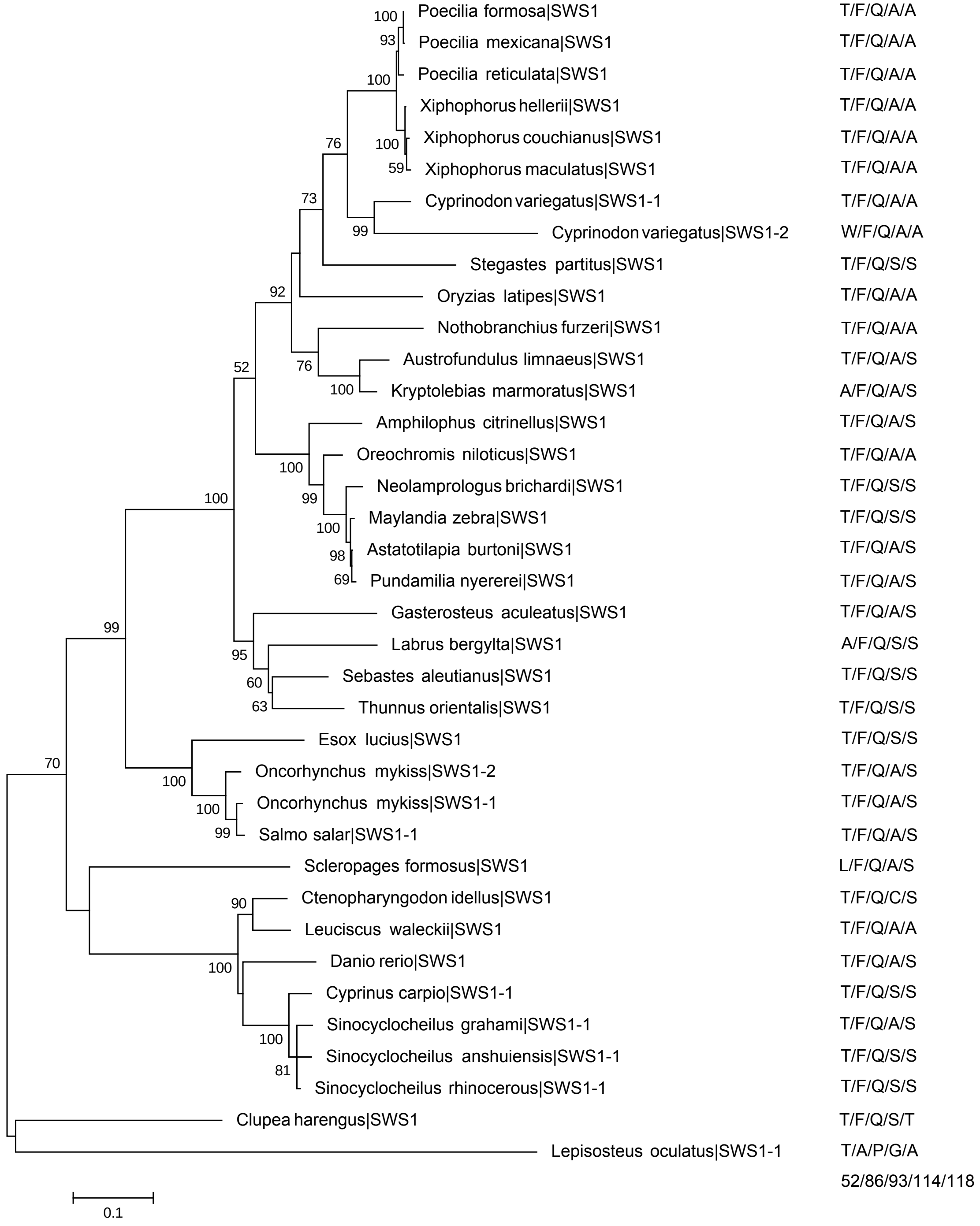

Supplementary Figure S4 Maximum likelihood phylogeny of the complete SWS1 genes. For each complete SWS1 gene, we also indicate its key tuning sites (52/86/93/114/118).

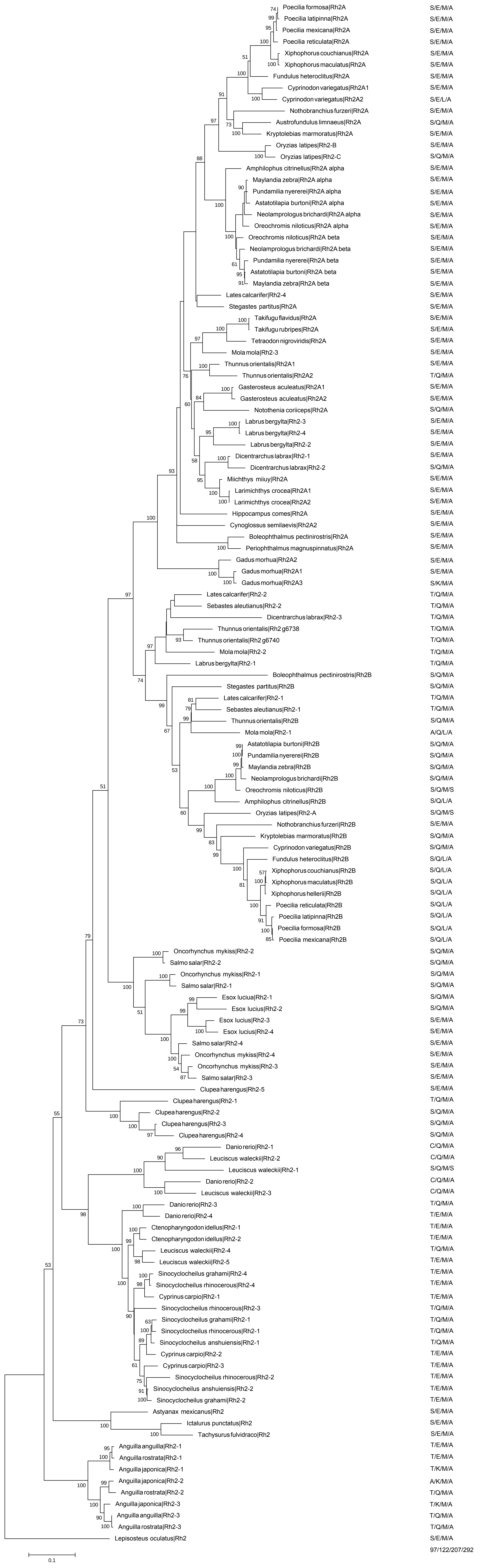

Supplementary Figure S5 Maximum likelihood phylogeny of the complete Rh2 genes.
For each complete Rh2 gene, we also indicate its key tuning sites (97/122/207/292).

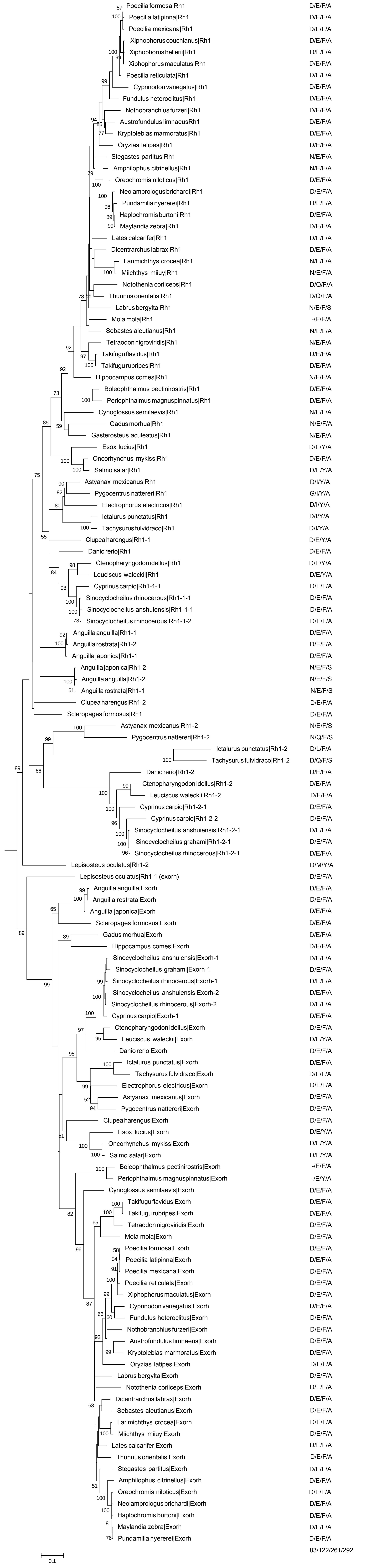

Supplementary Figure S6 Maximum likelihood phylogeny of the complete Rh1 genes. For each complete Rh1 gene, we also indicate its key tuning sites (83/122/261/292). A tuning site is labeled by "-" if its corresponding coding region was not sequenced.

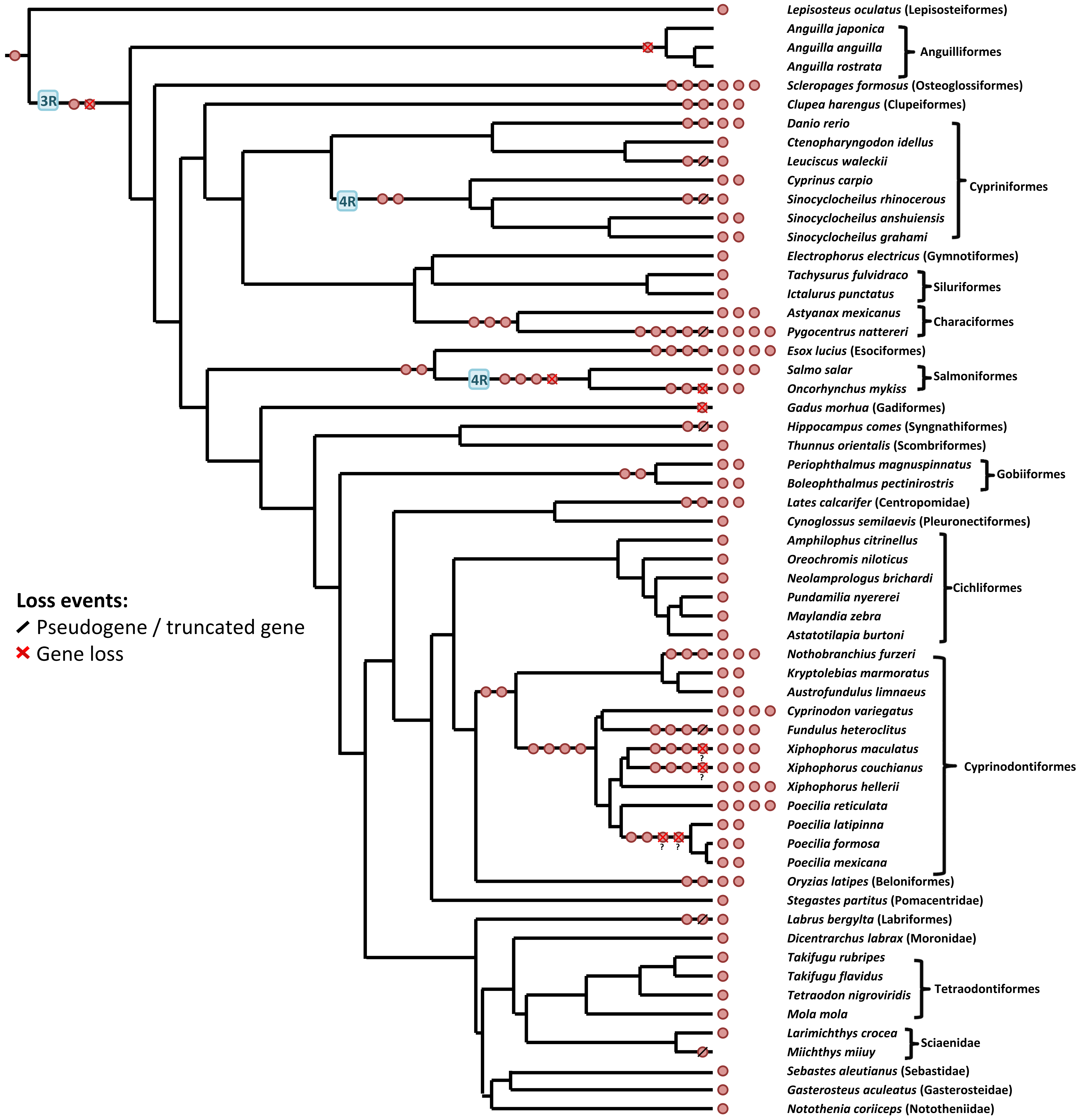

Supplementary Figure S7. The tree of copy number changes of LWS gene in the 59 ray-finned fish genomes. The gene symbols were defined in Fig. 1. For the ancestral states or lineages with gain/loss events, we labeled the predicted configuration at the corresponding positions on the reference tree.

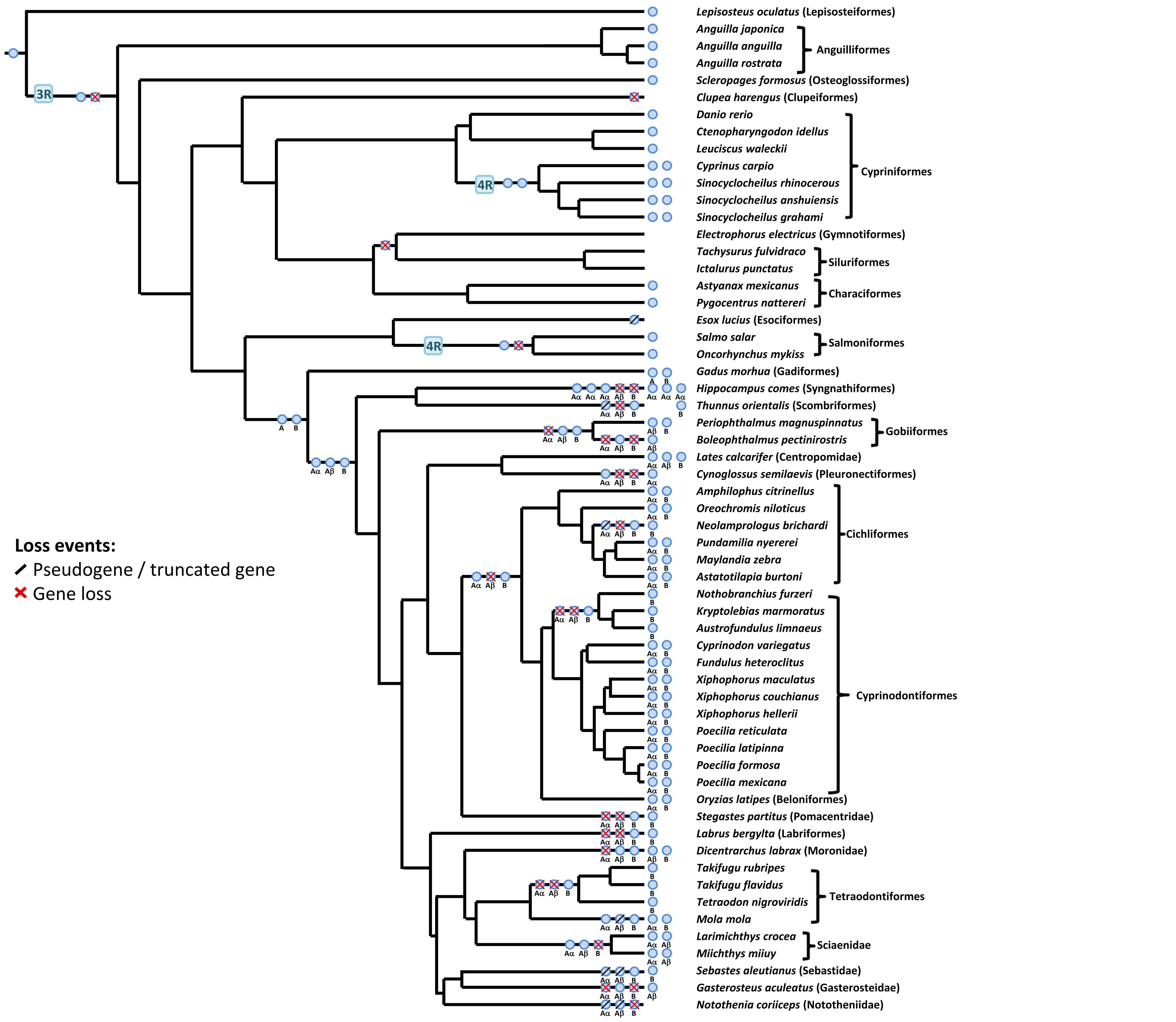

Supplementary Figure S8. The tree of copy number changes of SWS2 gene in the 59 ray-finned fish genomes. The gene symbols were defined in Fig. 1. For the ancestral states or lineages with gain/loss events, we labeled the predicted configuration at the corresponding positions on the reference tree.

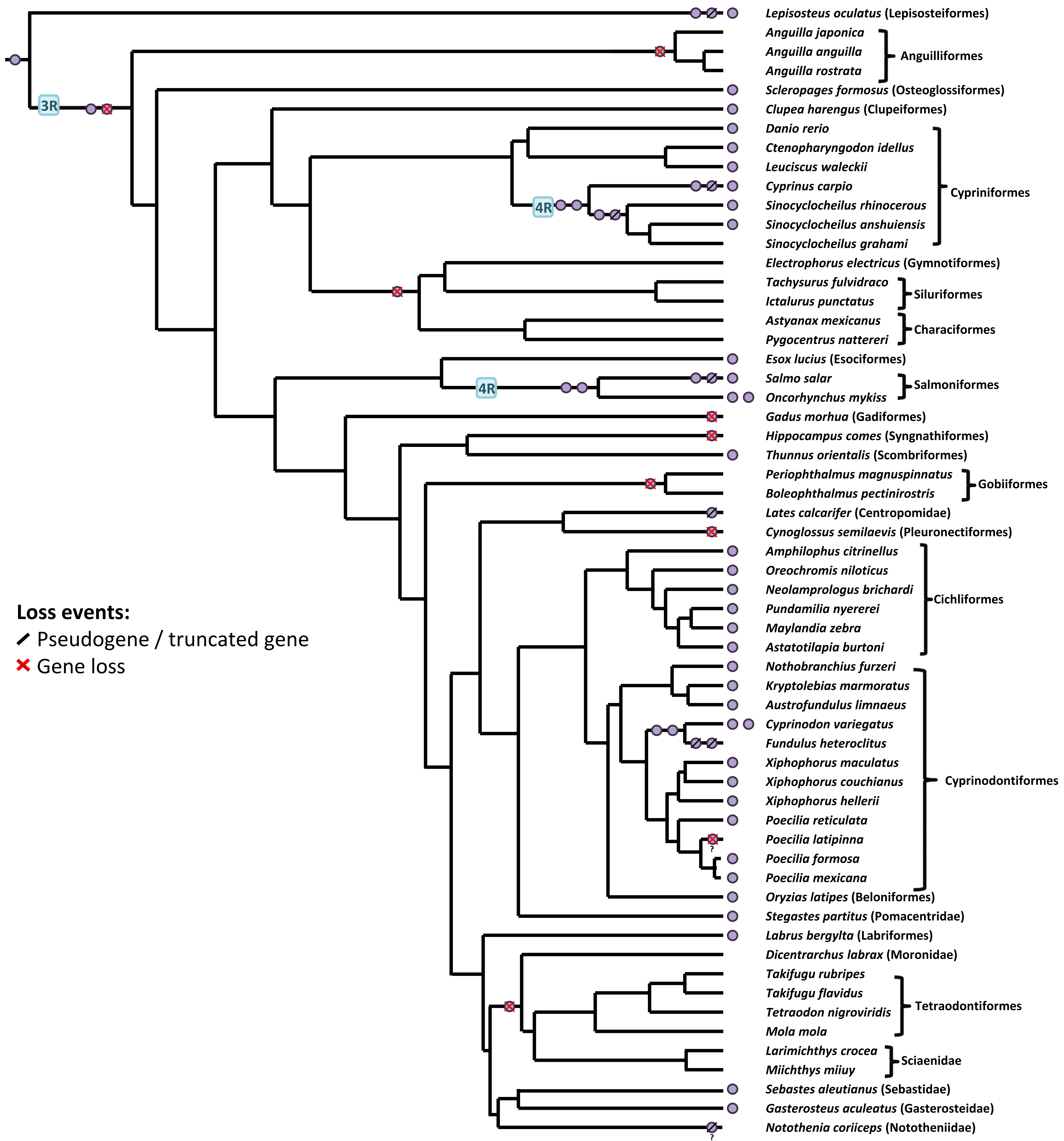

Supplementary Figure S9. The tree of copy number changes of SWS1 gene in the 59 ray-finned fish genomes. The gene symbols were defined in Fig. 1. For the ancestral states or lineages with gain/loss events, we labeled the predicted configuration at the corresponding positions on the reference tree.

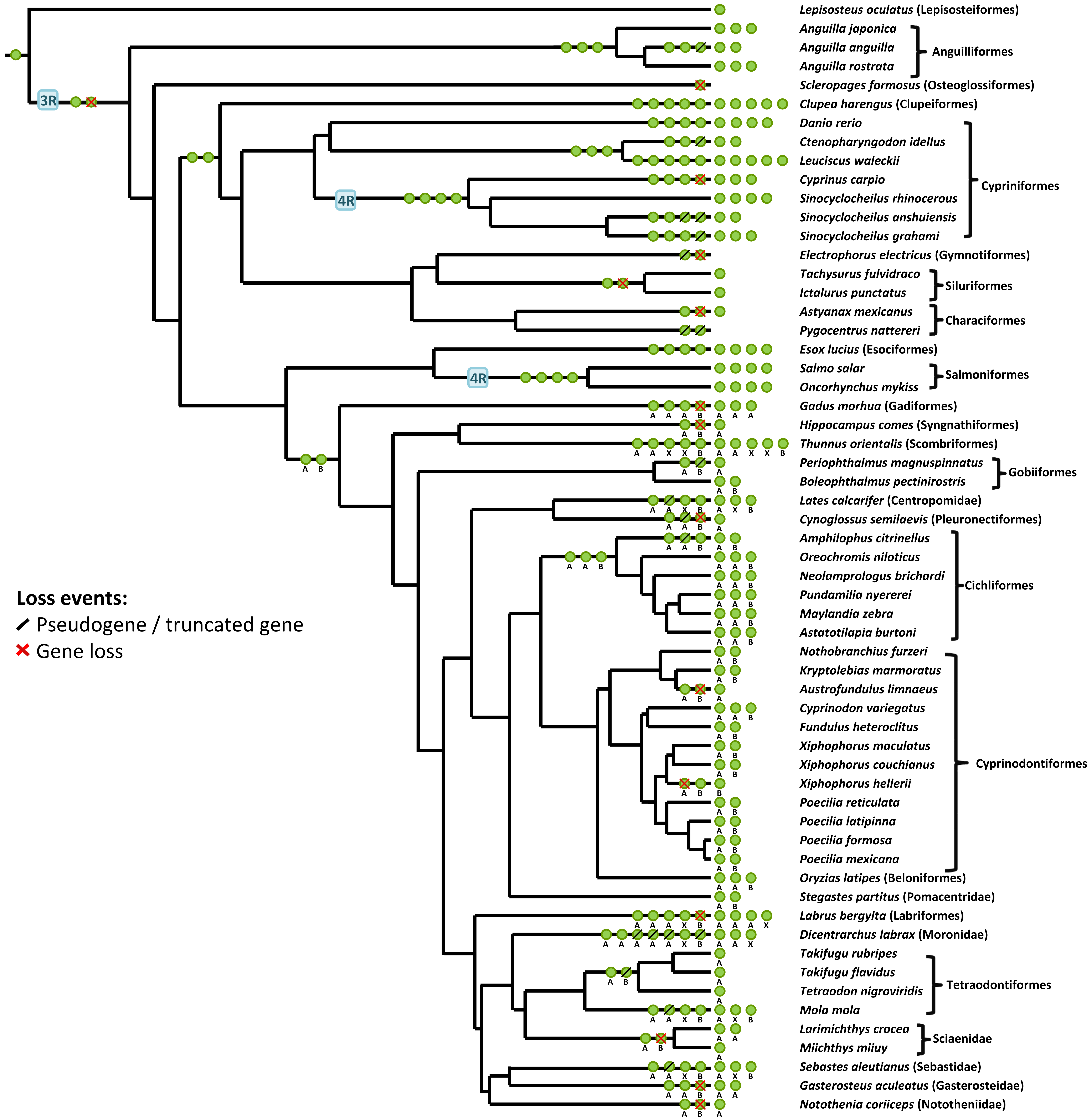

Supplementary Figure S10. The tree of copy number changes of Rh2 gene in the 59 ray-finned fish genomes. The gene symbols were defined in Fig. 1. For the ancestral states or lineages with gain/loss events, we labeled the predicted configuration at the corresponding positions on the reference tree.

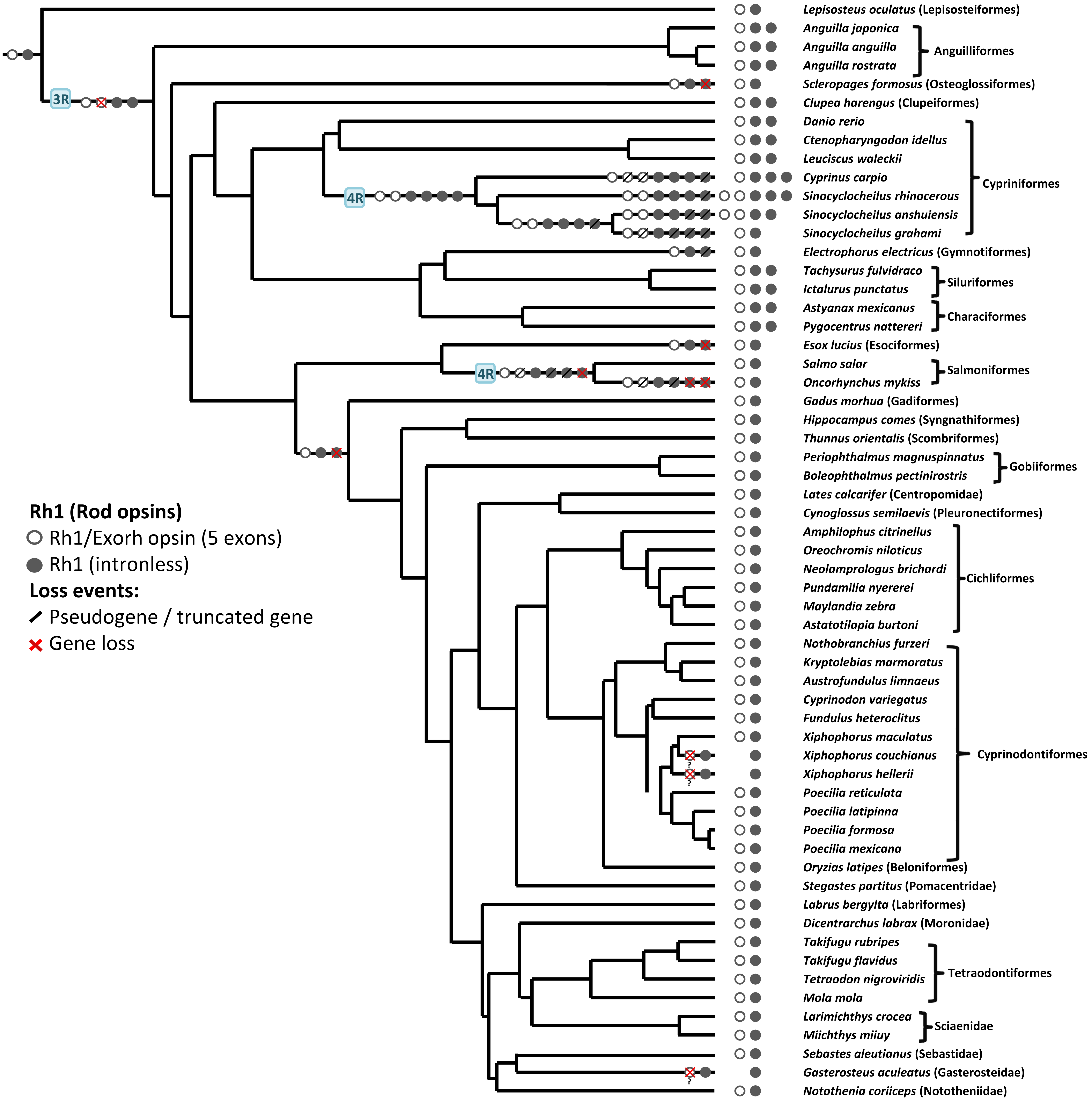

Supplementary Figure S11. The tree of copy number changes of Rh1 gene in the 59 ray-finned fish genomes. The gene symbols were defined in Fig. 1. For the ancestral states or lineages with gain/loss events, we labeled the predicted configuration at the corresponding positions on the reference tree.

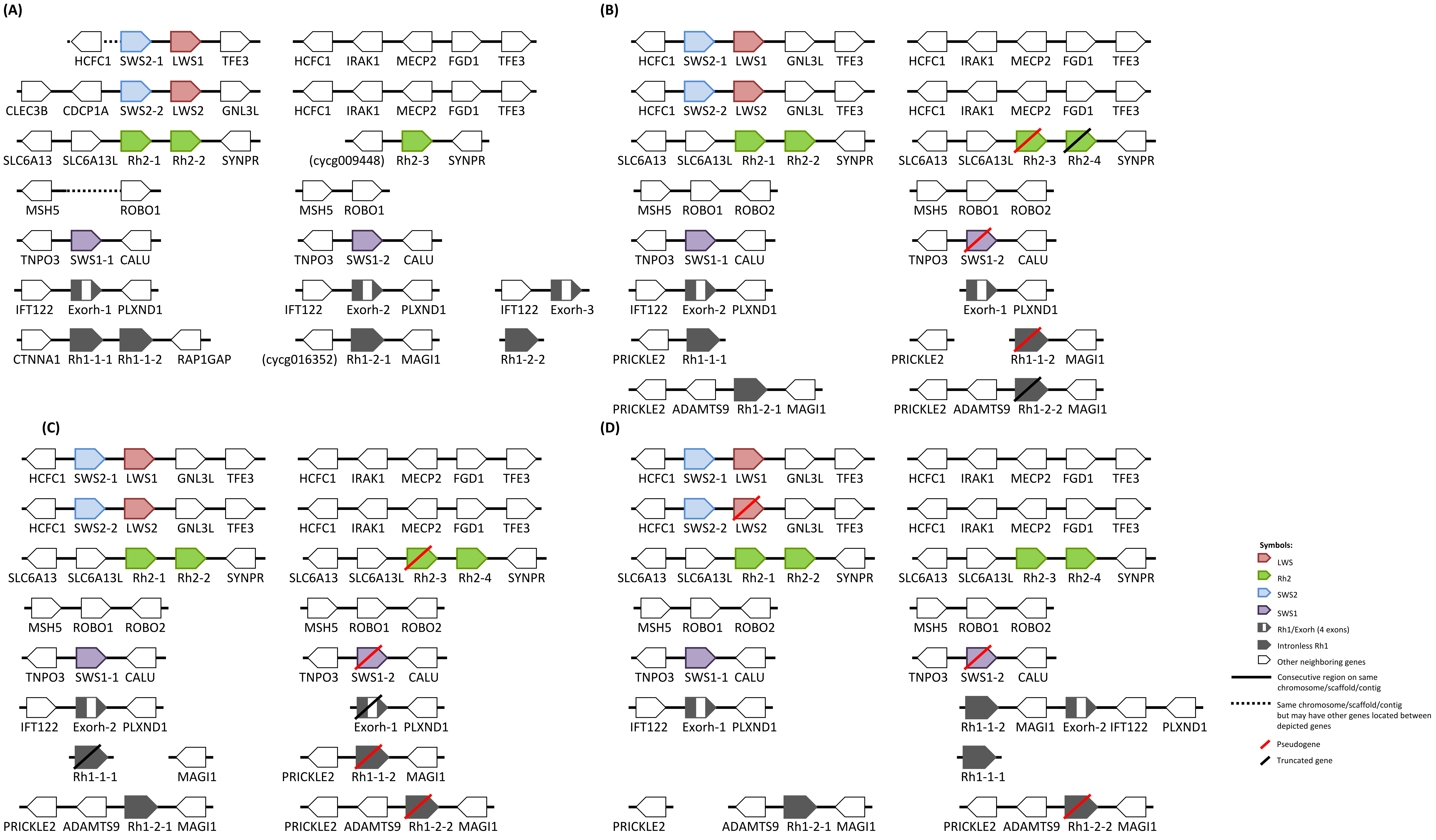

Supplementary Figure S12. The effect of 4R in *Cyprinus carpio* and *Sinocyclocheilus spp*. (Cypriniformes). The syntenies in (A) *Cyprinus carpio*, (B) *Sinocyclocheilus anshuiensis*, (C) *Sinocyclocheilus grahami* and (D) *Sinocyclocheilus rhinocerous* are shown. The genomic location of each synteny is summarized in Supplementary Table S4. The WGD event in these species doubled all the opsin gene syntenies. The genes which did not appear in the opsin gene syntenies in other species are as follows: CLEC3B (C-type lectin domain family 3 member B), CDCP1A (CUB domain containing protein 1a), CTNNA1 (catenin alpha 1), RAP1GAP (RAP1 GTPase activating protein) and RPB1 (DNA-directed RNA polymerase II subunit RPB1). For genes with unknown names, we indicate their gene ID in the corresponding annotation.

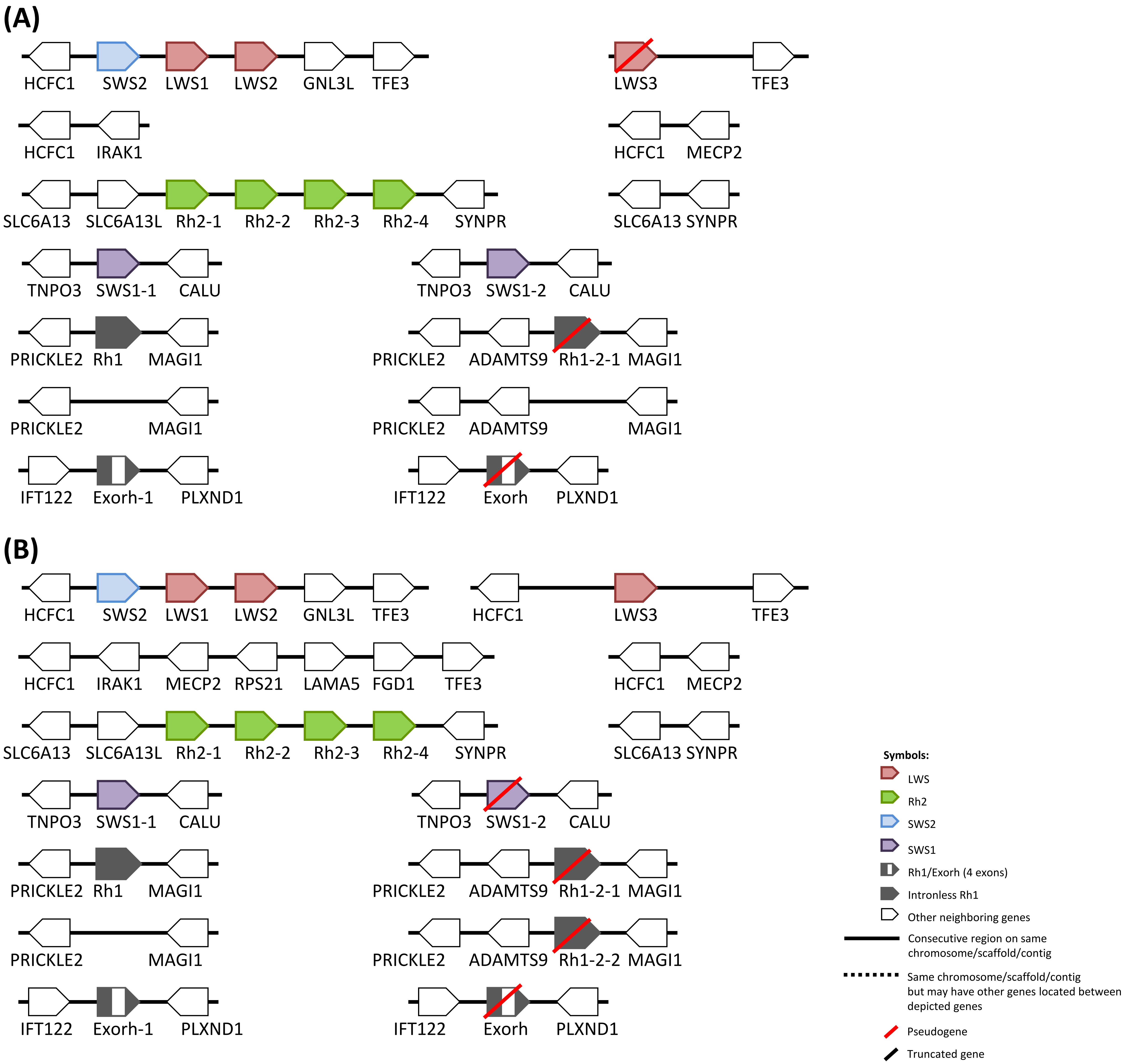

Supplementary Figure S13. The effect of 4R in Salmoniformes. The syntenies in (A) *Oncorhynchus mykiss* and (B) *Salmo salar* are shown. The genomic location of each synteny is summarized in Supplementary Table S4. The syntenies of intronless Rh1 and exorh were duplicated but the duplicated opsin genes became pseudogenes in both species. The newly duplicated SWS2-LWS synteny in both species contains the LWS3 gene; however, the LWS3 gene in *O. mykiss* is a pseudogene. The newly duplicated Rh2 synteny in both species presently contains only the typical neighboring genes such as SLC6A13 and SYNPR but no Rh2 genes. The newly duplicated SWS1 synteny presently contains 1 extra copy of the SWS1 gene; however, the extra copy in *S. salar* is a pseudogene. The genes which did not appear in the opsin gene syntenies in other species are RPS21 (40S ribosomal protein S21) and LAMA5 (laminin subunit alpha-5).
